# Design of High‐Performance Organic Semiconductors by Intra‐ and Intermolecular Charge Transfer Interaction

**DOI:** 10.1002/smsc.202500374

**Published:** 2025-09-18

**Authors:** Mozhgan Shahmirzaee, Hassan Alipour, Arthisree Devendran, Krzysztof Lyczko, Atsushi Nagai

**Affiliations:** ^1^ Next‐Generation Energy Systems Group Ensemble3‐ Centre of Excellence 01‐919 Warsaw Poland; ^2^ Laboratory for Spectroscopy, Molecular Modeling and Structure Determination Institute of Nuclear Chemistry and Technology 03‐195 Warsaw Poland

**Keywords:** capacitance, charge transfer, complex, conductivity

## Abstract

Charge transfer (CT) interactions have rarely been used to organize supramolecules and cross‐linked objects. However, the ever‐increasing understanding of CT interactions opens up new avenues for the design of innovative materials with tailored electronic properties. Herein, several molar ratios of highly crystalline π‐conjugated *n*%TCNQ@Sq‐1,6Py oligomers (*n* equal molar ratio of tetracyanoquinodimethane (TCNQ) to 1,6‐diaminopyrene (1,6Py) moiety) with simultaneously stable intra‐ and intermolecular CT mechanisms are prepared. As a result, the *π*‐conjugated 200%TCNQ@Sq‐1,6Py CT complex indicates stable intra‐ and intermolecular CT interactions resulting in extremely high electrical conductivity of 8.7 × 10^−2^ S cm^−1^ at room temperature, a charge‐distance capacitance of 70.62 F g^−1^ at the current density of 0.625 A g^−1^ which significantly increases to 968.7 F g^−1^ by doping of polyaniline (PANI) at a current density of 0.312 A g^−1^. Finally, it exhibits a capacitance retention of 70% of the initial specific capacitance after 1000 cycles at room temperature. This type of *π*‐conjugated oligomer CT complex can be used to improve existing CT‐based energy storage devices, such as capacitors.

## Graphical abstract

Here, π‐conjugated CT complexes are reported with stable inter‐intramolecular CT natures, including n%TCNQ@Sq‐1,6Py and *200*%TCNQ@Sq‐1,6Py/PANI composite. The *200*%TCNQ@Sq‐1,6Py and 200%TCNQ@Sq‐1,6Py/PANI indicate considerable electrical conductivity of 8.7 × 10^−2^ S cm^−1^ and high specific capacitance of 968.7 F g^−^¹, respectively, compared to the recent literature with a capacitance retention of 70%.

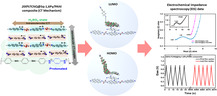

## 1 Introduction

Recent advances in electrode materials for energy storage mechanisms have encompassed a broad range of materials and design strategies.^[^ ^1^, ^2^ ^]^ Charge transfer (CT) in π‐conjugated organic complexes arises from the interaction between π‐donor and π‐acceptor units within the same molecule (intramolecular CT) or between separate molecules (intermolecular CT).^[^ ^3^, ^4^ ^]^ In the context of CT, the connection rate between the donor and acceptor units translates into a faster and more efficient CT process, which is crucial for CT applications, such as room‐temperature phosphorescence,^[^ ^5^, ^6^ ^]^ tunable emission probes,^[^ ^7^ ^]^ optical waveguides,^[^ ^8^ ^]^ molecular motors or actuators,^[^ ^7^ ^]^ organic photovoltaic materials,^[^ ^9^, ^10^ ^]^ two‐photon absorption,^[^ ^11^ ^]^ organic field‐effect transistors,^[^ ^12^, ^13^ ^]^ and white light generation.^[^ ^14^, ^15^ ^]^ The key challenge of CT research is to improve the rate of stable interactions between the donor and acceptor moieties, in particular, through the strategic selection of organic compounds for the purpose of optimizing charge transport and electrochemical performance.

Among the various molecular frameworks studied, pyrene and tetracyanoquinodimethane (TCNQ) have demonstrated promising CT properties by forming stable and conductive molecular assemblies.^[^ ^16^, ^17^, ^18^, ^19^ ^]^ In particular, 1,6‐diaminopyrene (1,6Py) exhibits a significant donor property (−4.78 eV) that is even stronger than tetrathiafulvalene (TTF), despite its low oxidation potential.^[^ ^20^ ^]^ These findings suggest that pyrene‐TCNQ‐based complexes (donor–acceptor) provide a strong foundation for developing high‐performance organic electronic materials due to their intrinsic π‐conjugated systems. Zhu et al. recently investigated, through theoretical research, the charge transport characteristics of mixed stack crystals using C_8_BTBT‐FnTCNQ and DMQtT‐F_4_TCNQs, and verified their potency as both hole and electron transporters.^[^ ^21^ ^]^ Dobrowolski et al. have shown that CT in the stacking pyrene–perylene‐TCNQ complex occurred more efficiently than in other TCNQ complexes with benzenoid hydrocarbons.^[^ ^22^ ^]^ Notably, fewer studies have focused on the oligomeric structure in the context of the CT mechanism, despite the potential relevance of molecules, like squaric acid (Sq), which is particularly intriguing in both organic and supramolecular chemistry due to its electron‐deficient π‐system and multiple hydrogen bond donor and acceptor sites. Each carbonyl group has a lone pair of electrons on the oxygen atom, making it a strong H‐bond acceptor. The negative charge in resonance forms is delocalized over the oxygen atoms, increasing the electron density on the oxygen atoms, making them more effective acceptors. In addition, Sq is planar and rigid, promoting predictable and strong interactions in supramolecular assemblies or crystal engineering, which allows for bidentate or bridging interactions, where two H‐bond donor groups can interact simultaneously with the two carbonyl oxygens.^[^ ^23^, ^24^ ^]^ In addition, some polymer compounds have been used to improve CT complex properties. Polyaniline (PANI) is one of the most extensively studied conducting polymers due to its unique chemical structure and redox‐active properties. The delocalized π‐electrons along the phenylene backbone facilitate electron mobility which allows efficient overlap with acceptor orbitals. PANI can easily donate electrons due to its accessible oxidation states, particularly in the emeraldine base form. This makes it adaptable to interact with various electron‐deficient acceptors (e.g., squaric acid, TCNQ, and so on).^[^ ^25^, ^26^ ^]^ Previous studies have evaluated theoretically or experimentally the performance of pyrene‐ or TCNQ‐based CT systems, however, thus far, it has not been investigated whether pyrene‐based oligomer‐TCNQ complex exhibits simultaneous intra‐ and intermolecular CT mechanisms.

**Figure 1:**
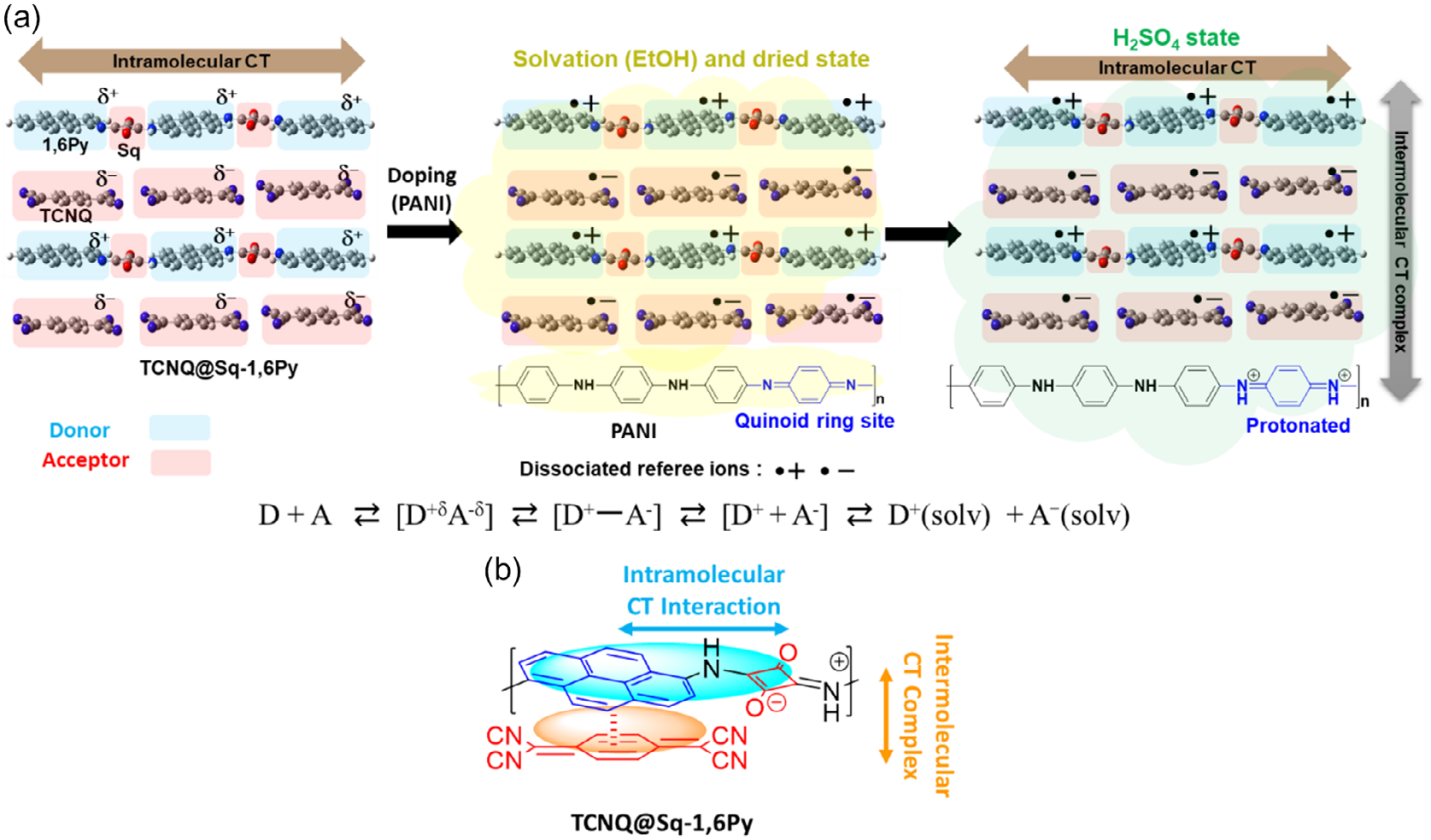
a) CT structure mechanism of *n*%TCNQ@Sq‐1,6Py and *n*%TCNQ@Sq‐1,6Py/PANI composite; red represents the acceptor moieties and blue represents the donor moieties. b) Schematic of representation of *n*%TCNQ@Sq‐1,6Py chemical reaction and molecular structure of *n*%TCNQ@Sq‐1,6Py.

Herein, we focus on the two CT mechanisms simultaneously observed in π‐conjugated organic complexes of *n*%TCNQ@Sq‐1,6Py and *n*%TCNQ@Sq‐1,6Py/PANI composite (**Figure** **1**): an intramolecular interaction between 1,6Py (donor) and squaric acid (Sq, acceptor); an intermolecular CT complex between 1,6Py (donor) and TCNQ (acceptor); and an additional intermolecular CT interaction between PANI (donor based on its quinoid/hydroquinone redox system) and TCNQ (acceptor).

When 1,6Py and TCNQ interact, electron density is partially transferred from the highest occupied molecular orbital (HOMO) to lowest unoccupied molecular orbital (LUMO) of pyrenediamine to the LUMO of TCNQ. This leads to the formation of a donor–acceptor complex stabilized by π–π stacking and electrostatic attraction. There are noncovalent interactions, including strong π–π stacking between the planar pyrene core and TCNQ and possible hydrogen bonding via —NH_2_ groups with cyano groups. In addition, 1,6Py–Sq oligomer as donor and acceptor are covalently linked in a repeating backbone → classic intramolecular CT. PANI also has alternating quinoid (=N—) and hydroquinone (—NH—) units. The redox‐active structure allows delocalized electron density along the backbone. In the emeraldine state, PANI can donate electrons from its π‐system and nitrogen lone pairs. Overall, the molecular interactions are π–π stacking between TCNQ and the aromatic segments of PANI, Coulombic stabilization between partially oxidized donor and reduced acceptor, and potential hydrogen bonding between PANI's amine units and TCNQ's–CN groups.

To enable intramolecular communication, the moieties are often covalently linked and have a high degree of π‐conjugation. In addition, strong intermolecular interactions occur between the donor and acceptor groups, such as hydrogen bonding and π‐stacking, which allow the formation of extremely stable complexes between molecules. Finally, the CT between PANI and TCNQ can be explained by the active mechanism in which PANI is protonated in the H_2_SO_4_ solution.

The simultaneous CT inter‐ and intramolecular interactions that occur in the 200%TCNQ@Sq‐1,6Py and 200%TCNQ@Sq‐1,6Py/PANI molecular structures lead to the formation of stable CT complexes with outstanding electrical conductivity and impressive specific capacitance, respectively. They demonstrate the potential of CT complexes between donor polycyclic aromatic hydrocarbons and acceptors based on a variety of CT interactions that lead to high electrical conductivity, low resistivity, and supercapacitance performance.

## 2 Results and Discussion

**Figure 2:**
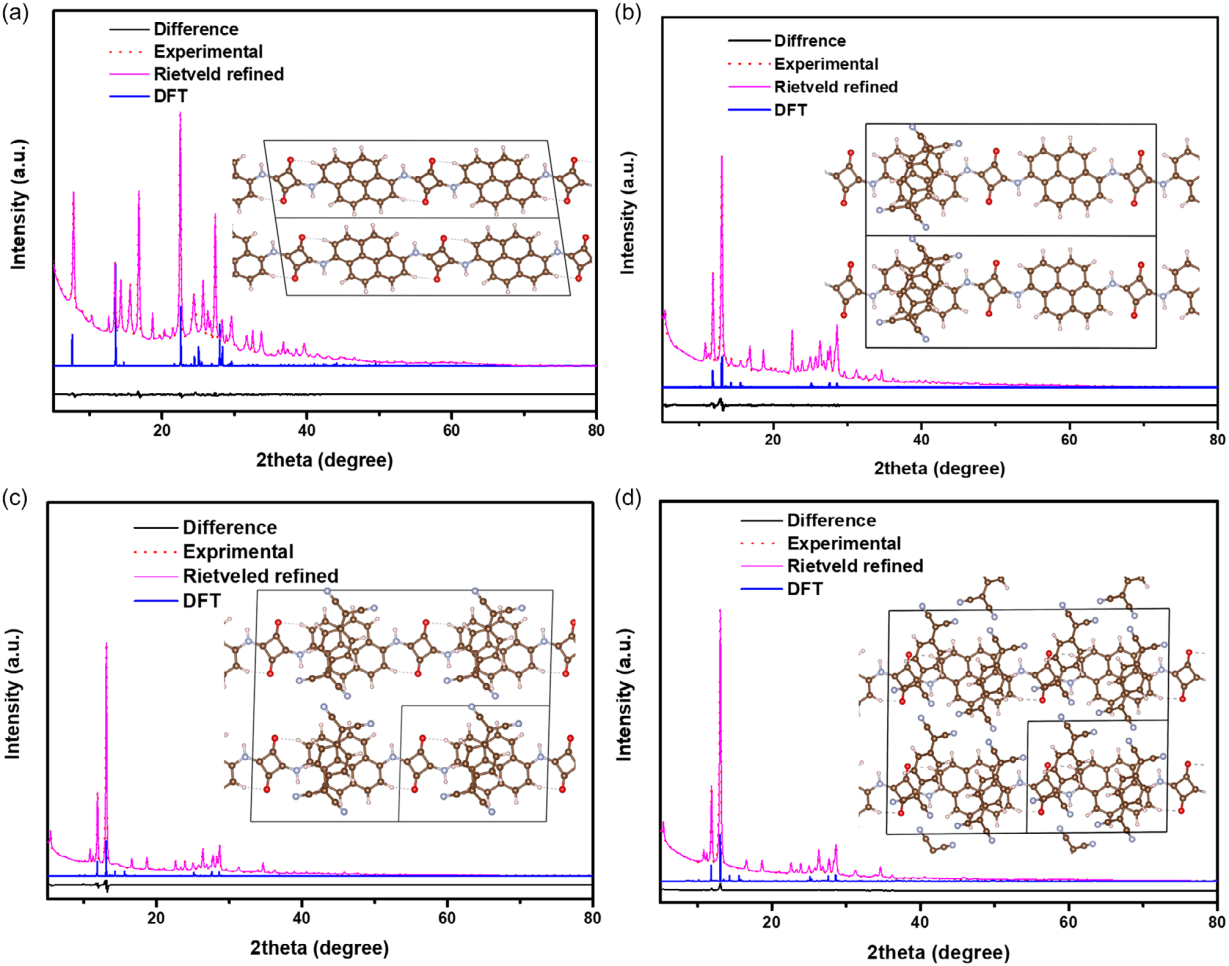
XRD data for the experimental versus Rietveld‐refined and simulated plus positions of atoms in crystal structures of *n*%TCNQ@Sq‐1,6Py with a) *n* = 0, b) 50, c) 100, and d) 200.

To validate the crystal structures of *n*%TCNQ@Sq‐1,6Py and the positions of atoms in them, we investigated both experimental and simulated X‐ray diffraction (XRD) patterns along with the model structure of CT complexes (**Figure** **2**a–d). It revealed that all synthesized CT structures are crystalline and consist of triclinic crystal structures made of TCNQ and 1,6Py fragments. Interestingly, further analysis of the CT complex experimental XRD patterns revealed that the dominant peak at 7.839° associated with 1,6Py disappeared. However, two new strong peaks at 11.7° and 13.06° successfully emerged from the hydrogen‐bonding interactions in squaramide with two more peaks at 27.4° and 28.6° which in turn came from π–π stacking interactions that finally confirm the successful production of CT complexes.^[^ ^27^, ^28^ ^]^ Meanwhile, the simulated XRD results achieved with density functional theory (DFT) calculations strongly correlate with the experimental data, clearly indicating the presence of prominent peaks in the structure. Interestingly, based on the model structures, strong intermolecular interactions allow self‐assembly into well‐aligned packing structures with plane‐to‐plane meeting of the TCNQ and Sq‐1,6Py moieties in the CT complex. The Supporting Information contains further crystallographic data in various unit cell descriptions (*n*%TCNQ@Sq‐1,6Py: *n* = 0, 50, 100, and 200) and lattice parameters in the triclinic (Table S1, Supporting Information). In addition, the measured XRD patterns of the structures are subjected to Rietveld refinement based on the optimized DFT structures, resulting in being in good agreement with experimental patterns as shown in Figure 2a–d. The predicted perfectly flat structure of the oligomer is subjected to deformation in the unicell angles, and changes in length occur mostly in the b lattice vector. Furthermore, the 200%TCNQ@Sq‐1,6Py/PANI composite experimental XRD pattern (Figure S1, Supporting Information) demonstrates major characteristic peaks belonging to PANI, proving its semi‐crystalline nature.

We also investigated the morphological nature and details of *n*%TCNQ@Sq‐1,6Py CT complexes with *n* = 0, 100, and 200 by transmission electron microscopy (TEM) images (Figure S2a–c, Supporting Information). The results revealed mesoporous structures in which some dark areas correspond to the additional TCNQ, validating the flak and layer structures of CT complexes. Interestingly, the higher and lower scales of 20 nm confirm oligomer and mesoporous structures, respectively. For further study of the images, scanning electron microscopy (SEM) was utilized to examine the microstructure and surface morphology of the crystalline CT complexes (Figure S3a–d, Supporting Information). As shown, the CT complexes form a densely packed, flake‐like structure several nanometers deep. Consequently, compact structures were found in which the agglomerations and dimensions increase with rising concentration of TCNQ from 115 ± 10 nm (*n* = 100) to 190 ± 12 nm (*n* = 200), confirming that increased TCNQ content promotes particle aggregation and growth, which in turn reduces the accessible surface area (Figure S3e–f, Supporting Information).

Furthermore, the nitrogen gas adsorption of *n* = 100 and 200%TCNQ@Sq‐1,6Py was measured to assess their pore size distribution, total pore volume, and specific surface area, which can affect charge transport and adsorption characteristics.^[^ ^29^ ^]^ According to the results (Figure S4a–b, Supporting Information), samples are categorized as type IV with an H3 hysteresis loop based on the IUPAC classification, suggesting a monolayer under high pressure, followed by a multilayer. Additionally, the Barret–Joyner–Halenda analysis indicates the mesoporous CT complexes as well, which aligns with the porosity findings from the TEM results. Following, the *n* = 100 and 200%TCNQ@Sq‐1,6Py displayed Brunauer, Emmett, and Teller surface area of 334.69 and 194.5 m^2^ g^−1^, also the average pore diameter of 4.40 and 5.22 nm, respectively. The decreased specific surface area in CT complexes is due to the aggregation phenomenon and increased dimensions of particles as revealed from SEM images.

To validate the bonding structure between atoms of CT complexes, we detail here our analysis of the Fourier transform infrared (FTIR) specifics of *n*%TCNQ@Sq‐1,6Py (Figure S5a, Supporting Information). The FTIR analysis confirms the coordination between the oligomer atoms and TCNQ atoms, separately, which show characteristic peaks at 834 cm^−1^ (C—H stretching); 1507, 1529, and 1560 cm^−1^ (squaramide C=O bond vibration); 1595 cm^−1^ (C=C stretching);^[^ ^20^ ^]^ 2157 and 2185 cm^−1^ (C≡N vibration bands of TCNQ); and 3191 and 3337 cm^−1^ (N—H stretch in the amine group), confirming the CT complex formation. Consequently, C—NH—C and C=N—C bridge bonds are created as the oxidative process moves forward, creating an organic complex. In addition, to gain a more detailed understanding of CT in the 200%TCNQ@Sq‐1,6Py/PANI composite, we also measured its FTIR analysis (Figure S5b, Supporting Information). The —C≡N stretching vibration bands appeared in 2224, 2185, and 2157 cm^−1^ belonging to TCNQ°, TCNQ^−1^, and TCNQ^−1^ states, respectively. As a result, the natural state of TCNQ (TCNQ°) confirms the inactive TCNQ/PANI structure, meaning no CT reaction between PANI and TCNQ molecules solved in the EtOH solution. However, the wavenumbers shifted (39 and 67 cm^−1^) confirm the charged states of TCNQ (TCNQ^−1^) led to CT between Sq‐1,6Py and TCNQ in 200%TCNQ@Sq‐1,6Py and 200%TCNQ@Sq‐1,6Py/PANI composite successfully.^[^ ^30^ ^]^

To get insight into the elemental nature of *n*%TCNQ@Sq‐1,6Py CT complex, we examined X‐ray photoelectron spectroscopy (XPS) analysis. Figure S6, Supporting Information, shows the high‐resolution XPS spectra (O 1s, C 1s, and N 1s) of *n*%TCNQ@Sq‐1,6Py. XPS analysis of the C1s region reveals prominent peaks at 284.2 eV C consistent with C species in TCNQ.^[^ ^31^ ^]^ The high‐resolution N1s spectrum at 400.9 eV corresponds to N in the TCNQ, the coordinated N atom in the pyrene group, and N in the C—NH—C linkage.^[^ ^32^ ^]^ Furthermore, the O1s spectrum at 531.6 eV is attributed to oxygen in the squaric ring.^[^ ^33^ ^]^ The Supporting Information in Table S2 provides the chemical composition of *n*%TCNQ@Sq‐1,6Py.

Consistent with a more structural investigation, UV spectroscopy was utilized to observe the electronic transitions in the *π*‐conjugated *n*%TCNQ@Sq‐1,6Py as illustrated in Figure S7, Supporting Information. The UV–Vis spectra of the *50*, 100, and 200%TCNQ@Sq‐1,6Py CT complexes exhibit a broad absorption range of 1500–2000 nm. The intensity of the pyrene absorption peak at 530 nm decreased and the intensity of the CT complex absorption at the region of 1500–2000 nm considerably increased with increasing TCNQ/Sq‐1,6Py molar ratio. In support of the experimental analysis, simulated studies of UV–vis spectrum of 1,6Py (175, 215, and 337 nm), TCNQ (411 nm), and Sq‐1,6Py (530 nm) obtained from DFT calculation (Figure S8a‐c, Supporting Information) show good agreement with the measurement results in Figure S7, Supporting Information.

### 2.1 DFT Calculation Studies

**Figure 3:**
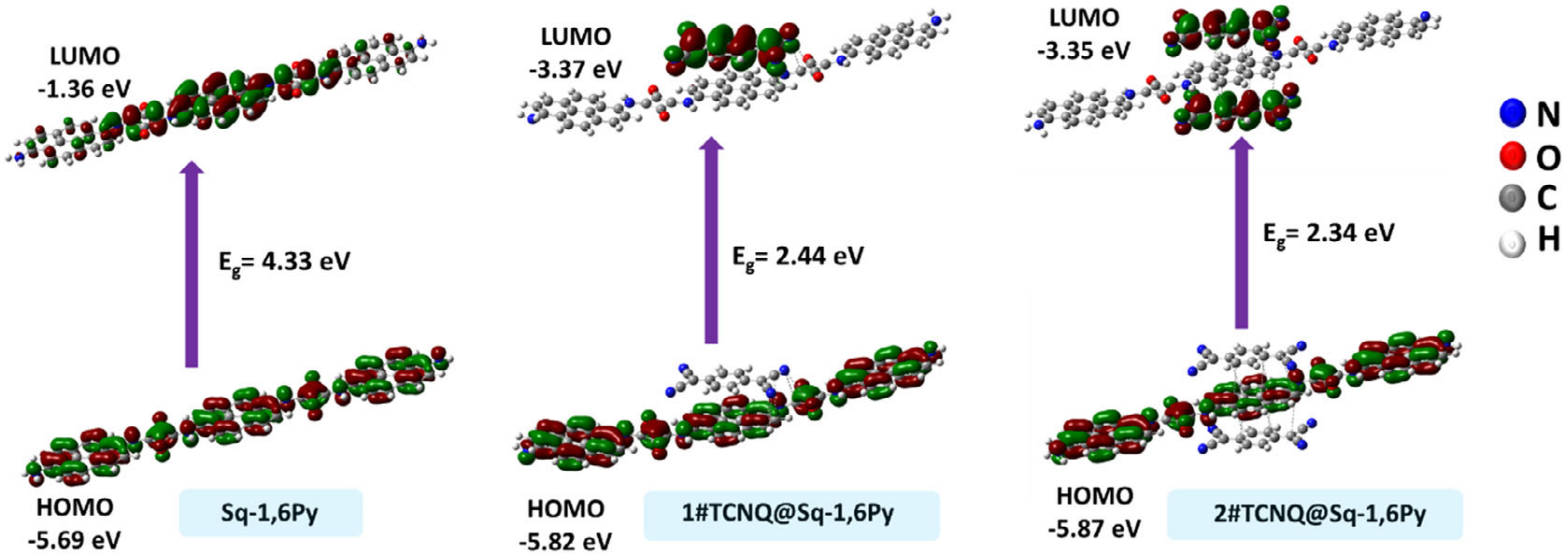
Molecular orbitals of TCNQ/Sq‐1,6Py complex. HOMO and LUMO calculation with different numbers of TCNQ molecules (1 and 2); red and green colors describe the positive and negative phases, respectively.

To gain insight into the electronic structure of TCNQ molecule interacted to pristine Sq‐1,6Py molecule, we carried out DFT computations (**Figure** **3**) on a fragment Sq‐1,6Py with different numbers of TCNQ molecules (1 and 2) using Gaussian 16. The Supporting Information contains further the structures obtained from DFT (Figure S9, Supporting Information). The molecular orbitals of TCNQ/Sq‐1,6Py complex exhibits covalent bonding and π‐conjugation, which is facilitated by π‐orbital overlap between adjacent atoms and electron delocalization across the molecular framework. Moreover, adding an amino group increases the HOMO energy level as a result of a strong resonance (+R) effect.^[^ ^34^ ^]^ The *E*_g_ of the 1#TCNQ/Sq‐1,6Py and 2#TCNQ/Sq‐1,6Py CT complexes decreased from 2.44 to 2.34 eV as a quantitative effect of the presence of TCNQ in the CT complexes. Therefore, the incorporation of the TCNQ molecule into the CT complex enhances the CT process by introducing a π‐donor–acceptor system that facilitates electron donation. This observation is well supported by the UV–Vis spectral data.

We also studied the orbital effects of Sq‐1,6Py oligomer, TCNQ, and PANI molecules from DFT calculations to determine the CT mechanism between donor and acceptor fragments. As shown in Figure S10, Supporting Information, the HOMO and LUMO distributions in the solid state with a band gap of 2.59 eV, do not suggest significant CT between PANI and TCNQ. In contrast, the dominant CT mechanism in the TCNQ/Sq‐1,6Py/PANI composite arises from the interaction between the Sq‐1,6Py donor and the TCNQ acceptor. However, based on the band gap analysis, an alternative CT mechanism is proposed involving the HOMO‐1 and LUMO due to the close energy levels of HOMO (−5.79 eV) and HOMO‐1 (−5.83 eV). The calculated HOMO–LUMO energy gap is 2.63 eV, resulting from the lower energy level of HOMO‐1, suggesting a CT interaction between the PANI donor and the TCNQ acceptor. Finally, the CT between Sq‐1,6Py donor → TCNQ acceptor ← PANI donor is considered as the main mechanism with the highest contribution to electron transport.

### 2.2 Electrochemical Properties

**Figure 4:**
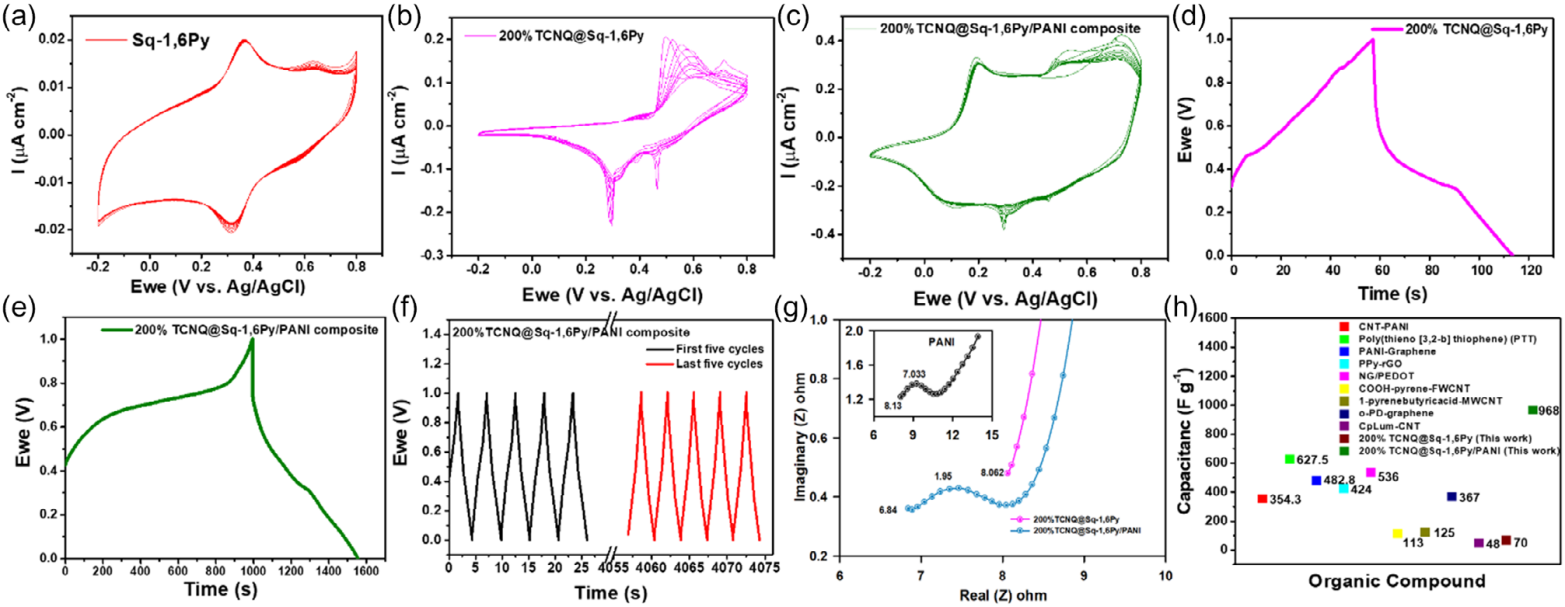
Electrochemical display of the electrodes in a three‐electrode system in the aqueous electrolyte (0.5 M H_2_SO_4_). a) C–V curve of Sq‐1,6Py. b) C–V curve of 200%TCNQ@Sq‐1,6Py. c) C–V curve of 200%TCNQ@Sq‐1,6Py/PANI composite. d) Charge–discharge profile of 200%TCNQ@Sq‐1,6Py. e) Charge–discharge profile of 200%TCNQ@Sq‐1,6Py/PANI composite at a current density of 0.312 A g^−1^. f) Cycling stability of 200%TCNQ@Sq‐1,6Py/PANI composite for 1000 charge‐discharge cycles. g) Nyquist impedance plots of PANI, 200%TCNQ@Sq‐1,6Py, and 200%TCNQ@Sq‐1,6Py/PANI composite. h) Capacitance of some organic compounds based on the literature.

Interestingly, we evaluated the electrochemical performance of the 200%TCNQ@Sq‐1,6Py‐ and 200%TCNQ@Sq‐1,6Py/PANI composite‐based electrodes using galvanostatic charge–discharge (GCD) and cyclic voltammetry (CV) measurements. The development of 200%TCNQ@Sq‐1,6Py and its composite with PANI aims to synergize CT capabilities, redox activity, and pseudocapacitive behavior, thereby improving charge storage performance and electrical conductivity. **Figure** **4**a–c shows the CV curves of the Sq‐1,6Py, 200%TCNQ@Sq‐1,6Py, and 200%TCNQ@Sq‐1,6Py/PANI composite electrodes with a scanning rate of 50 mV s^−1^. The Supporting Information contains further information on the CV curves of TCNQ, PANI, TCNQ/PANI, and 200%TCNQ@Sq‐1,6Py/PANI (10 and 80 mV^−1^ scan rate) (Figure S11a–e, Supporting Information). A close examination reveals a good agreement between GCD curves and CV data. The results reveal the redox‐active nature and capacitive response of each component. The CV curves (Figure 4a–c) demonstrate an increasingly prominent current density and broad shape from Sq‐1,6Py to 200%TCNQ@Sq‐1,6Py/PANI, highlighting enhanced redox contributions and pseudocapacitance. Overall, the composite supramolecular complex undergoes a reversible redox process during supercapacitor operation, where both the donor and acceptor components contribute to charge storage through complementary mechanisms. So, PANI donor undergoes well‐established redox transitions.

(1) $${\text{PANI}}^{0}⇌{\text{PANI}}^{+}+e^{-}$$

This contributes to pseudocapacitance through fast and reversible electron/proton exchange at redox‐active nitrogen centers. In addition, TCNQ is a strong π‐acceptor, capable of accepting one or two electrons through

(2) $${\text{TCNQ}+e}^{-}⇌{\text{TCNQ}}^{\cdot -}{\text{ and TCNQ}}^{\cdot -}+e^{-}⇌{\text{TCNQ}}^{2-}$$

These reversible reduction steps contribute significantly to the overall capacitance via Faradaic processes. During charging, the donor is oxidized and TCNQ is reduced, enhancing the CT interaction. Upon discharging, electron flow reverses, but the supramolecular structure remains largely intact due to π–π stacking and persistent noncovalent interactions.

GCD results (Figure 4d–e) support these findings, with the composite achieving a remarkably high capacitance of 968.7 (± 2, SD = 2) F g^−1^ (current density of 0.312 A g^−1^) compared to 70.6 (±2, SD = 2) F g^−1^ for 200%TCNQ@Sq‐1,6Py complex alone (current density of 0.625 A g^−1^). This demonstrates that integrating PANI with TCNQ@Sq‐1,6Py significantly boosts electrochemical performance, which comes from combining the pseudocapacitive behavior of PANI with the redox activity and CT capabilities of TCNQ and Sq‐1,6Py. The improvement comes from dual CT interactions—intermolecular (TCNQ and 1,6Py) and intramolecular (Sq and 1,6Py) and the high surface area of PANI facilitating electrolyte access and rapid ion diffusion.^[^ ^29^ ^]^ Furthermore, the results showed the capacitance of 418.75 F g^−1^ at 0.312 A g^−1^ for TCNQ/PANI composite (Figure S11f, Supporting Information). This indicates that ≈57% of the total capacitance in our PANI‐doped system arises from the intrinsic contribution of the preorganized crystalline CT backbone, which facilitates more efficient electron transport and ion diffusion compared to the binary PANI/TCNQ system. The , Supporting Information contains further GCD data of the composite in various current densities of 0.285 A g^−1^ and 0.75 A g^−1^ (Figure S11g–i, Supporting Information). The results showed that other current densities are not stable when compared to the optimal results.

In addition, the composite retains 70% of its original capacitance over 1,000 cycles (Figure 4f and Figure S12, Supporting Information), reflecting good structural and electrochemical stability. The conjugated network and CT framework likely prevent degradation during high‐rate operation. It is noticeable that the CT interactions in our system are not solely dependent on transient redox states but are stabilized by strong π–π stacking and complementary electronic properties between the donor and acceptor. While redox events may modulate charge distribution, the supramolecular architecture remains intact due to persistent noncovalent interactions. This is supported by our electrochemical data (CV, GCD, and cycling performance), which show excellent reversibility and stability, indicating no dissociation of the CT complex upon discharge.

Electrochemical impedance spectroscopy (EIS) data (Figure 4g) show a decrease in CT resistance ($R_{ct}$ = 1.95 (± 2, SD = 0.02) Ω) for the composite compared to pristine PANI (7.033 (± 0.6, SD = 0.6) Ω), confirming enhanced electronic conductivity. This aligns with conductivity values increasing dramatically with TCNQ content from 5.5 × 10^−6^ S cm^−1^ (0% TCNQ) to 8.7 × 10^−2^ S cm^−1^ (200% TCNQ), indicating that CT interactions promote charge delocalization and mobility due to the excellent TCNQ charge carrier (TCNQ°) source and strong 1,6‐Py electron donor characteristics. TCNQ^−^ as electroactive anions with anionogen groups incorporated in PANI enhance the electroactivity of the polymer, while exhibiting its electrochemical activity.^[^ ^35^ ^]^

For further studies, we investigated the EIS of 200%TCNQ@Sq‐1,6Py and 200%TCNQ@Sq‐1,6Py/PANI composite under UV light (365 nm) and heating, separately (Figure S13, Supporting Information). Under UV and thermal treatment, the CT composite maintains conductivity with a reduced $R_{\text{ct}}$, suggesting stability and enhanced charge mobility under stimulus. This response is attributed to increased excitation and release of localized carriers in the CT framework.^[^ ^36^ ^]^ When compared to other organic supercapacitor materials (Figure 4h), our composite with the power density of 310.40 W kg^−1^ and energy density of 134.5 Wh kg^−1^ significantly outperforms many carbon‐based hybrids (e.g., CNT‐PANI,^[^ ^37^ ^]^ PANI‐Graphene,^[^ ^38^ ^]^ PPy‐rGO,^[^ ^39^ ^]^ CpLum‐CNT,^[^ ^40^ ^]^ few‐walled carbon nanotubes (FWCNT),^[^ ^41^ ^]^ multi‐walled carbon nanotubes (MWCNT),^[^ ^42^ ^]^ both in capacitance and conductivity. This underscores the effectiveness of CT complexation and PANI integration.

In keeping with the charge transport properties, we also measured the electrical conductivity of the CT complex. Interestingly, based on the results, *n*%TCNQ@Sq‐1,6Py with *n* = 0, 50, 100, and 200 exhibits outstanding electrical conductivity amounts of 5.5 × 10^−6^, 2.4 × 10^−4^, 1.29 × 10^−3^, and 8.7 × 10^−2^ S cm^−1^, and mobility values of 2.74 × 10^−2^, 3.49 × 10^−2^, 3.51 × 10^−2^, and 4.41 × 10^−2^ m^2^ (V s)^−1^, respectively. In particular, the electrical conductivity of 200%TCNQ@Sq‐1,6Py is approximately ten times of magnitude higher than that of 100%TCNQ@Sq‐1,6Py due to the synergistic effects of both inter‐ and intramolecular CT. The Sq core enforces ordered donor–acceptor stacking, enhancing π–π orbital overlap, while the dense TCNQ packing boosts charge carrier density through multiple intermolecular CT interactions per donor unit. This cooperative stabilization of both CT types‐unlike in classical systems, such as TTF–TCNQ‐, directly underpins the high conductivity (8.7 × 10^−2^ S cm^−1^). The strong intermolecular CT interaction between the 1,6Py donor and TCNQ acceptor and intramolecular CT interaction between 1,6Py donor and Sq acceptor generally leads to the creation of a delocalized electronic state, which reduces the energy barriers for charge movement, thereby enhancing charge carrier mobility and contributing to the overall conductivity of the material. In addition, narrower band gaps enhance charge carrier transport by reducing the energy barrier for conduction. Finally, we presented a brief description comparing the electrical conductivity of our CT complexes with other organic materials (Figure S14, Supporting Information).

As confirmation data and further supporting information, the calculated band gap data through the DFT method, UV–Vis, and CV curves are reported in Table S3, Supporting Information, showing good agreement between different measurement methods.

## 3 Conclusion

In conclusion, our study demonstrates the successful synthesis and comprehensive characterization of highly crystalline *π*‐conjugated *n*%TCNQ@Sq‐1,6Py complexes with *n* = 0, 50, 100, and 200 molar ratios. Detailed structural and photophysical analyses, supported by theoretical DFT calculations provide valuable insights into the design process of organic semiconductors in which simultaneous intra‐ and intermolecular CT interactions are leveraged to achieve enhanced conductivity and stability. The electrochemical performance assessments further highlight the potential of CT complexes in energy conversion applications. Notably, the 200%TCNQ@Sq‐1,6Py complex exhibits significant electrical conductivity (8.7 × 10^−2^ S cm^−1^) and capacitance (70 F g^−1^), which is further enhanced with PANI compositing (968.7 F g^−1^) and the resistance is efficiently reduced (1.95 Ω)—these parameter results are the highest compared to the data available in the recent literature on organic materials. These findings reveal new avenues for optimizing CT processes, thereby contributing to the advancement of innovative materials for electronic and energy conversion technologies. Based on our studies, we believe that utilizing simultaneous intra‐ and intermolecular CT interactions between *π*‐conjugated oligomer CT complexes can result in the construction of energy storage devices, like capacitors, with extremely high capacitance and low resistance.

## 4 Experimental Section

4.1

4.1.1

##### Materials Characteristics

Using a conventional 2*θ* range of 5°–80° and Cu Kβ radiation operating at 40 kV and 30 mA, an X‐ray diffractometer (Rigaku SmartLab 3 kW Diffractometer, Rigaku Corporation, Tokyo, Japan) was used to analyze the crystalline structure of the samples. The microstructure and surface morphology of the samples were estimated using high‐resolution TEM (HRTEM, Talos F200X G2 at 200 kV) and field emission SEM (FESEM, Zeiss Auriga, Jena, Germany). To find the pore volume and pore size distribution, nitrogen adsorption–desorption isotherms were measured at 77 K using the Micro200 system model with a power of 300 W and a voltage range of 100–240 V. Prior to testing, the samples were degassed in a vacuum for one hour at 150 °C to exclude any water. The surface areas within the range of 0.007–1.0 were computed in terms of relative pressure (P/P0). In addition, FTIR spectra were recorded using KBr pellets on a Thermo Scientific Nicolet iS10 FTIR spectrometer, covering a wavelength range of 400–4000 cm^−1^. Furthermore, XPS spectra were collected on ULVAC‐PHI VersaProbe 4 equipment. Moreover, the electrical conductivity of prepared pellet samples (*n*%TCNQ@Sq‐1,6Py, *n* = 0, 50, 100, and 200) under 10 bar pressure was measured on an electrochemical potentiostat (VSP‐300, BioLogic) at room temperature. Using the following formula, the electrical conductivity (σ) of samples was determined

(3) $$\sigma =\frac{L}{\rho \times A}$$

where *σ* is the inverse of resistivity (S cm^−1^), *R* is the electrical resistance of a uniform specimen of the material, and *L* and *A* are the specimen length (cm), and the cross‐sectional area of the specimen (cm^2^), respectively.

Additionally, CV at 50 mV^−1^ scan rate and GCD on the electrochemical potentiostat at room temperature were used to assess the electrochemical performances of the CT complexes. The measurements were carried out in 0.5 M H_2_SO_4_ electrolyte solution. The active components were drop cast onto the working electrode (glassy carbon working electrode (GCE)), while Ag/AgCl and a platinum wire functioned as the counter and reference electrodes, respectively. First, the following stock solutions were prepared; 4 mg of the samples (TCNQ, Sq‐1,6Py, PANI, and 200%TCNQ@Sq‐1,6Py) dispersed in 500 microliter ethanol and ultrasonicated for 1 min before coating on the GCE. From the stock solution, 2 microliters of TCNQ, Sq‐1,6Py, PANI, 200%TCNQ@Sq‐1,6Py were drop cast on the GCE for the electrochemical analysis, separately. The prepared 200%TCNQ@Sq‐1,6Py/PANI composite sample was measured under identical circumstances. All data in the average amount were reported after three repetitions.

Using the following formula, the electrode specific capacitance ($C_{\text{sp}}$) was determined

(4) $$C_{\text{sp}}=\frac{I\times Dt}{m\times \Delta V}$$

where *m* is the mass of active material (g), Dt is the discharge time (s), $C_{\text{sp}}$ denotes the specific capacitance of the modified electrode (F g^−1^), I presents the applied current during the discharge process (A), and ΔV is the change in potential (V). In addition, capacitance retention (CR) of the electrode was calculated using the following equation

(5) $$CR=\frac{C_{n}}{C_{1}}\times 100$$

where $C_{1}$ is the specific capacitance of the first cycle (F g^−1^) and $C_{n}$ is the specific capacitance of the last cycle (F g^−1^).

In addition, the electrical resistance (solution resistance: $R_{s}$ and CT resistance: $R_{\text{ct}}$) of PANI, TCNQ/PANI, TCNQ/ Sq‐1,6Py, 200%TCNQ@Sq‐1,6Py, and 200%TCNQ@Sq‐1,6Py/PANI were measured at room temperature using the cell with EIS R_1_+Q_2_/R_2_/W_2_ Randle circuit, the current range of 1 Hz–1 MHz, and 20 mV voltage. Subsequently, samples were prepared for analysis in the same way as mentioned above for the capacitance measuring. Furthermore, the stability of the films under light was evaluated by exposing them to UV–Vis irradiation (365 nm) for 20 and 40 min. Similarly, thermal stability was assessed by measuring the electrical resistance of the samples at various temperatures (25, 40, 60, and 80 °C).

##### DFT Studies

DFT and time‐dependent DFT calculations were applied to study theoretical investigations of model compounds to provide a more comprehensive knowledge of the structure and photophysical behavior of the *n*%TCNQ@Sq‐1,6Py CT complex. In the initial stage, the CT complexes were optimized using the Gaussian 16 W.^[^ ^20^ ^]^ With the use of the hybrid exchange‐correlation functional by the Coulomb‐attenuating method (CAM‐B3LYP) and the basis set level of 6–31 G(d,p), unrestricted formulation, the geometries of all chemical radical species were fully optimized without symmetry constraints.^[^ ^21^ ^]^ Subsequently, DFT calculations were carried out to determine the unit‐cell structure of the CT complex.^[^ ^22^ ^]^ As a result, the structural properties were accurately described in a way that was compatible with XRD observations. This helped to clarify atomistic configurational preferences and offered new information about functional CT complexes. These calculations were carried out via the plane‐wave Vienna ab initio simulation package (VASP) employing the Perdew–Burke–Ernzerhof^[^ ^23^ ^]^ functional, while the projector augmented wave method was implemented to probe the core‐electron interactions.^[^ ^24^ ^]^ In self‐consistent computations, the cutoff energy level of 500 eV for volume relaxation, and default values were used for structural relaxation in all cases.

##### Materials

Tetracyanoquinodimethane (TCNQ, 98%, Abcr company, Karlsruhe, Germany), and 1,6‐diaminopyrene (1,6Py, 98%, Abcr company, Karlsruhe, Germany), squaric acid (Sq, 99%, Sigma‐Aldrich, Gillingham, UK), and polyaniline (PANI, emeraldine base, 98%, Abcr company, Karlsruhe, Germany) were used as received. Anhydrous toluene (99.5%, Merck company, Taufkirchen, Germany), 1‐butanol (Chempur, Katowice, Poland), and Methanol (99.8%, Warchem, Warsaw, Poland) were prepared without any further purification.

##### Materials: Preparation of n%TCNQ@Sq‐1,6Py

The *n*%TCNQ@Sq‐1,6Py (*n* = 0, 50, 100, 200) was prepared according to the synthesis method by the controlled chemical route. The different amounts of TCNQ (0.05 mmol, 0.1 mmol, and 0.2 mmol) were added separately to three glass flasks (50 mm). Then, the same amounts of 1,6‐Py (0.1 mmol) and Sq (0.1 mmol) were added to each of the mentioned glass flasks (50 mm). Subsequently, a mixture of toluene (7 mL) and 1‐butanol (3 mL) was then added to the above flask. For 48 h, the initial mixture of materials was agitated at 400 rpm and 100 °C in a nitrogen environment. The reaction mixture was then allowed to reach room temperature. The *n*%TCNQ@Sq‐1,6Py CT complexes were purified by dropwise precipitation in methanol (100 mL) and collected by filtration. The final products were obtained after drying under reduced pressure and vacuum.

##### Materials: Preparation of 200%TCNQ@Sq‐1,6Py/PANI Composite

First, the following stock solutions were prepared; 4 mg of the samples (PANI and 200%TCNQ@Sq‐1,6Py) were dispersed in 500 microliter of ethanol and ultrasonicated for 1 min before being applied to the GCE for the electrochemical analysis (200%TCNQ@Sq‐1,6Py: PANI, 1:1 ratio). From the stock solution, 1 microliter suspension of 200%TCNQ@Sq‐1,6Py in ethanol was added to the working electrode (GCE) in a layer method and allowed to dry completely, and then 1 microliter suspension of PANI in ethanol was dropped on the dried previous layer. After the deposition of each layer, the samples were dried at room temperature for a specific time of 15 min and under a fume hood to ensure uniform film formation before proceeding to the next layer. This was repeated twice to study the effect of stacked layers under consideration (capacitance and resistance). In addition, the GCE measuring of TCNQ/PANI was studied in the same condition to make a composite layer.

## Supporting Information

Supporting Information is available from the Wiley Online Library or from the author.

## Conflict of Interest

The authors declare no conflict of interest.

## Author Contributions

**Mozhgan Shahmirzaee**: formal analysis (lead); methodology (lead); writing original draft (lead); writing—review and editing (equal). **Hassan Alipour**: data curation (lead); writing—review and editing (supporting). **Arthisree Devendran**: formal analysis (supporting). **Krzysztof Lyczko**: formal analysis (supporting). **Atsushi Nagai**: supervision (lead); writing—review and editing (lead).

## Supporting information

Supplementary Material

## Data Availability

The data that support the findings of this study are available in the supplementary material of this article.
