# Supplementary material for: Design of High‐Performance Organic Semiconductors by Intra‐ and Intermolecular Charge Transfer Interaction
Source: Small Sci. 2025 Sep 18;5(11):2500374. doi: 10.1002/smsc.202500374 (PMC12622481; doi:10.1002/smsc.202500374)
Supplement: Supplementary file 1 — Supplementary Material [file SMSC-5-2500374-s001.pdf]

Supplementary Information

Design of High-Performance Organic Semiconductors by Intra- and Intermolecular Charge Transfer Interaction

Mozhgan Shahmirzaee [a], Hassan Alipour [a], Arthisree Devendran [a], Krzysztof Lyczko [b], Atsushi Nagai [a,*]

[a] Next-Generation Energy Systems Group, , Ensemble3- Centre of Excellence, Warsaw, Poland
[b] Institute of Nuclear Chemistry and Technology, Warsaw, Poland

*Corresponding Author's Email: atsushi.nagai@ensemble3.eu

The obtained lattice parameters in the triclinic description are a =26.10, b = 7.29 Å and c = 4.20Å, α = 81.48°,β= 88.44°, γ= 98.18° for the Sq-1,6Py, a = 26.14, b = 7.34 Å and c = 10.13Å, α = 95.18°,β= 89.93°, γ= 90.01° for the *50*%TCNQ@Sq-1,6Py, a=13.06, b = 7.59811Å and c = 10.40Å, α = 101.18°,β= 91.75°, γ= 89.33° for the *100*%TCNQ@Sq-1,6Py, and a=13.05, b = 8.60 Å and c = 10.44 Å , α = 96.11°,β= 91.21°, γ= 89.16° for the *200*%TCNQ@Sq-1,6Py.

Table S1. Crystallographic data for the structures in various unit cells (*n*%TCNQ@Sq-1,6Py: *n*= 0, 50, 100, and 200).

Parameter	0%TCNQ@Sq-1,6Py	50%TCNQ@Sq-1,6Py	100%TCNQ@Sq-1,6Py	200%TCNQ@Sq-1,6Py
Empirical Formula	2($C_4H_2O_4$), 2($C_{16}H_{12}N_2$)	$C_{12}H_4N_4$, 2($C_4O_4H_2$), 2($C_{16}N_2H_{12}$)	$C_{12}H_4N_4$, $C_4O_4H_2$, $C_{16}N_2H_{12}$	2($C_{12}H_4N_4$), $C_4O_4H_2$, $C_{16}N_2H_{12}$
Composition	C40 N4 O4 H20	C52 N8 O4 H24	C32 N6 O2 H14	C44 N10 O2 H18
Formula weight	620.6112	824.798	514.493	718.679
Crystal system	triclinic	triclinic	triclinic	triclinic
Space group	P1	P1	P1	P1
Unit cell dimensions				
a (Å)	26.10247	26.13784	13.05874	13.04779
b (Å)	7.28814	7.34262	7.59811	8.59968
c (Å)	4.19768	10.12917	10.40176	10.43596
α (°)	81.4829	95.1822	101.1773	96.1070
β (°)	88.4386	89.9341	91.7481	91.2071
γ (°)	98.1863	90.0034	89.3320	89.1610
Volume (Å^3)	780.75	1936.04	1012.02	1163.99
Density (g/cm^3)	1.31994	0.70745	0.84422	1.0253
Z(molecules per cell)	4	4	2	2
θ range for data collection (°)	2.51-23.91	2.61-25.31	2.52-27.22	2.52-27.27
wR_2	0.0513	0.0722	0.0742	0.0754

In addition, the structural features of PANI and *200*%TCNQ@Sq-1,6Py/PANI composite were investigated by XRD measurement. As shown in Figure S1, major characteristic peaks with 2θ values at 15.26, 17.77, 19.99, 23.67, 26.35, 30.79, 33.60, and 40.84° belong to PANI confirms semi-crystalline nature. In addition, comparing the XRD pattern of *200*%TCNQ@Sq-1,6Py/PANI composite with that of *200*%TCNQ@Sq-1,6Py and PANI reveals that the new peaks appeared at 26.3° as a characteristic peak PANI [1]; this indicates the successful composition of *200*%TCNQ@Sq-1,6Py/PANI composite.

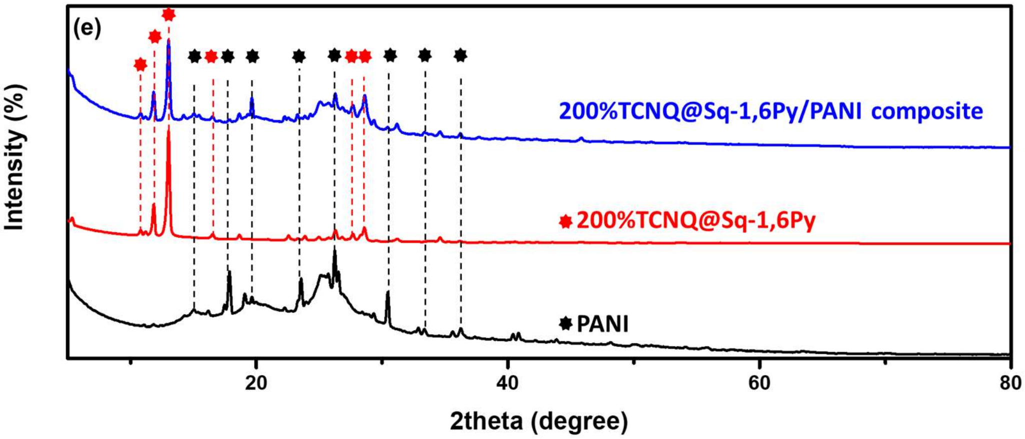

Figure S1. XRD pattern of PANI, *200*%TCNQ@Sq-1,6Py and *200*%TCNQ@Sq-1,6Py/PANI composite.

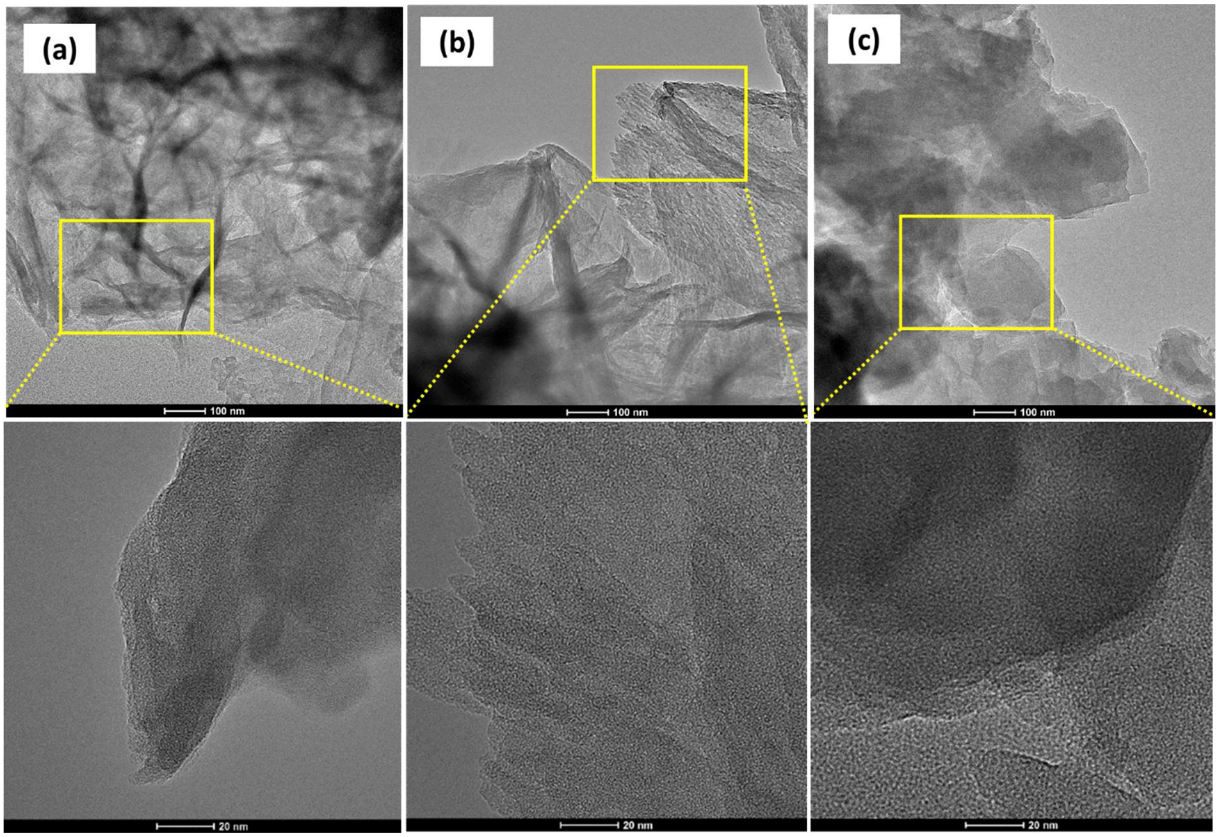

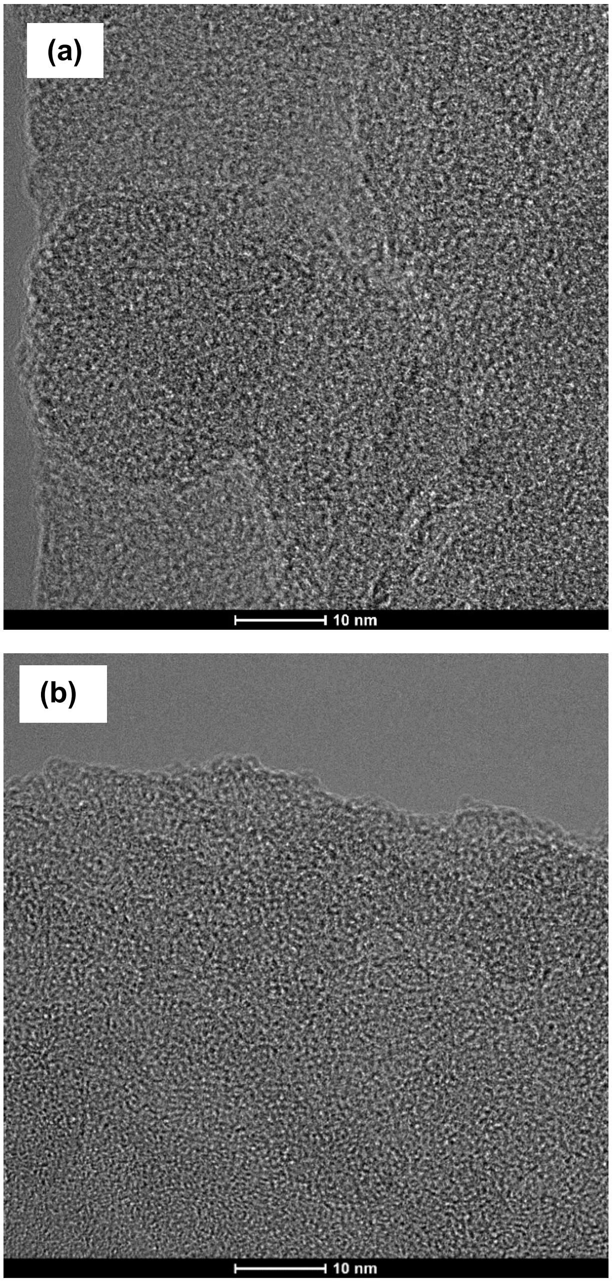
(a)
10 nm
(b)
10 nm

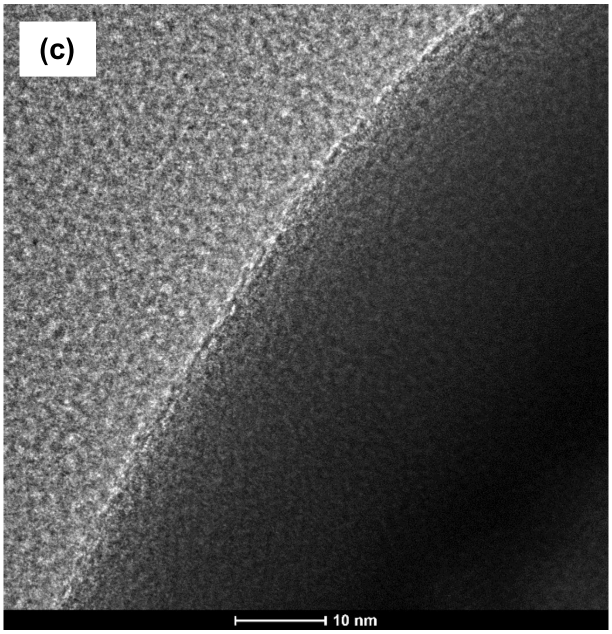

Figure S2. TEM results of *n*%TCNQ@Sq-1,6Py. a) *n*= 0. b) *n*= 100. c) *n*= 200 in different magnifications.

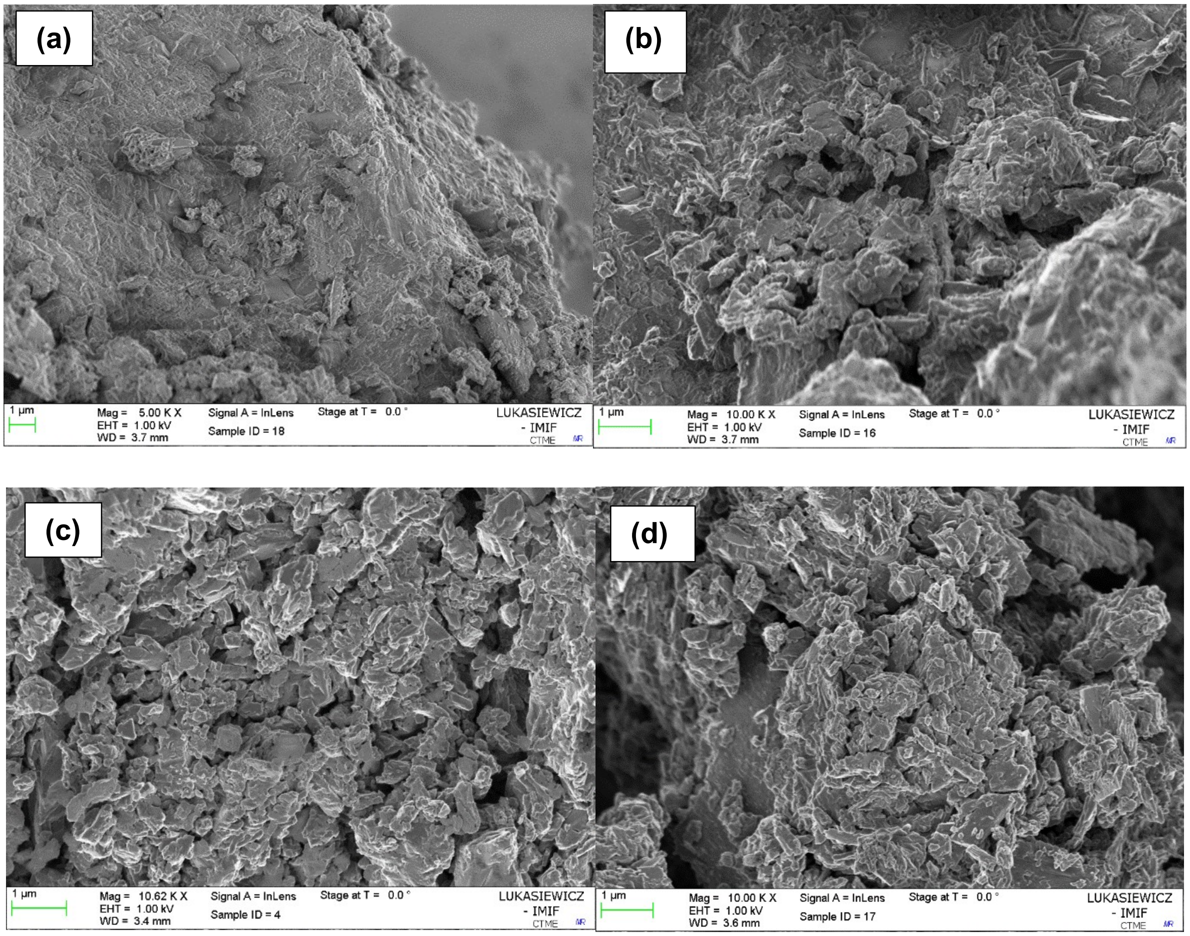

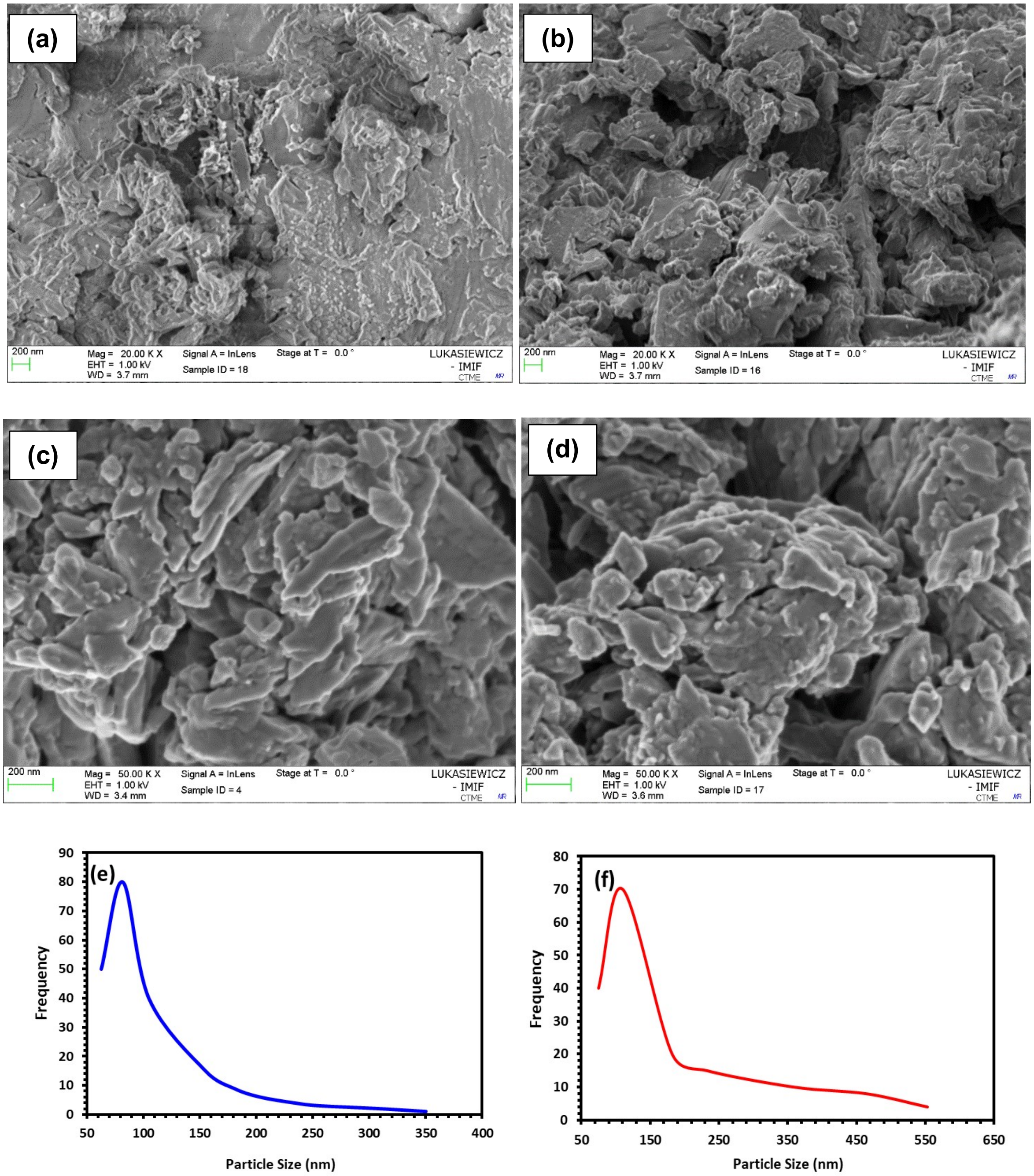

Figure S3. SEM micrographs of *n*%TCNQ@Sq-1,6Py. a) *n*= 0. b) *n*= 50. c) *n*= 100. d) *n*= 200. e) particle size distribution of *100*%TCNQ@Sq-1,6Py. f) particle size distribution of *200*%TCNQ@Sq-1,6Py.

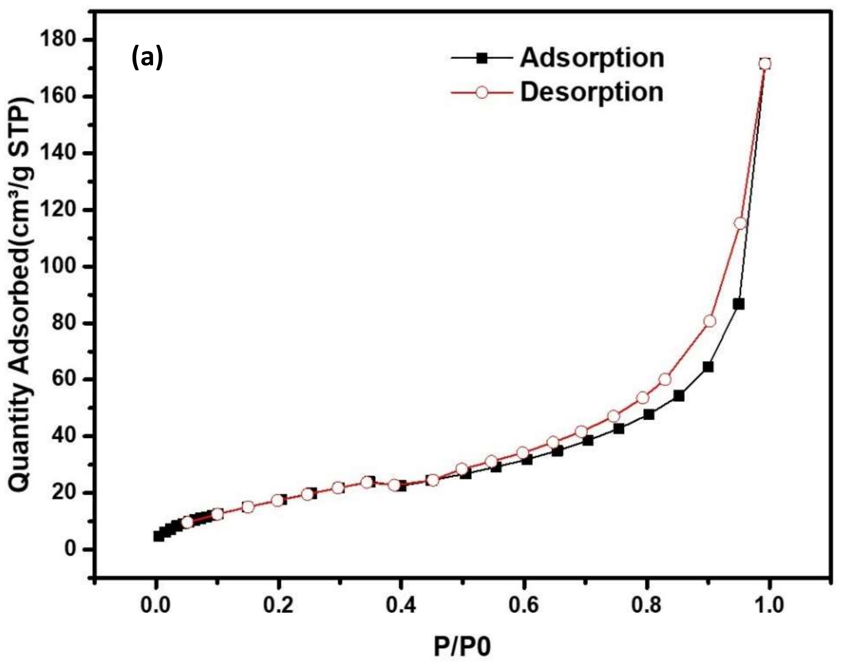

(a)
Adsorption
Desorption
Quantity Adsorbed(cm³/g STP)
P/P0
0.0
0.2
0.4
0.6
0.8
1.0
0
20
40
60
80
100
120
140
160
180

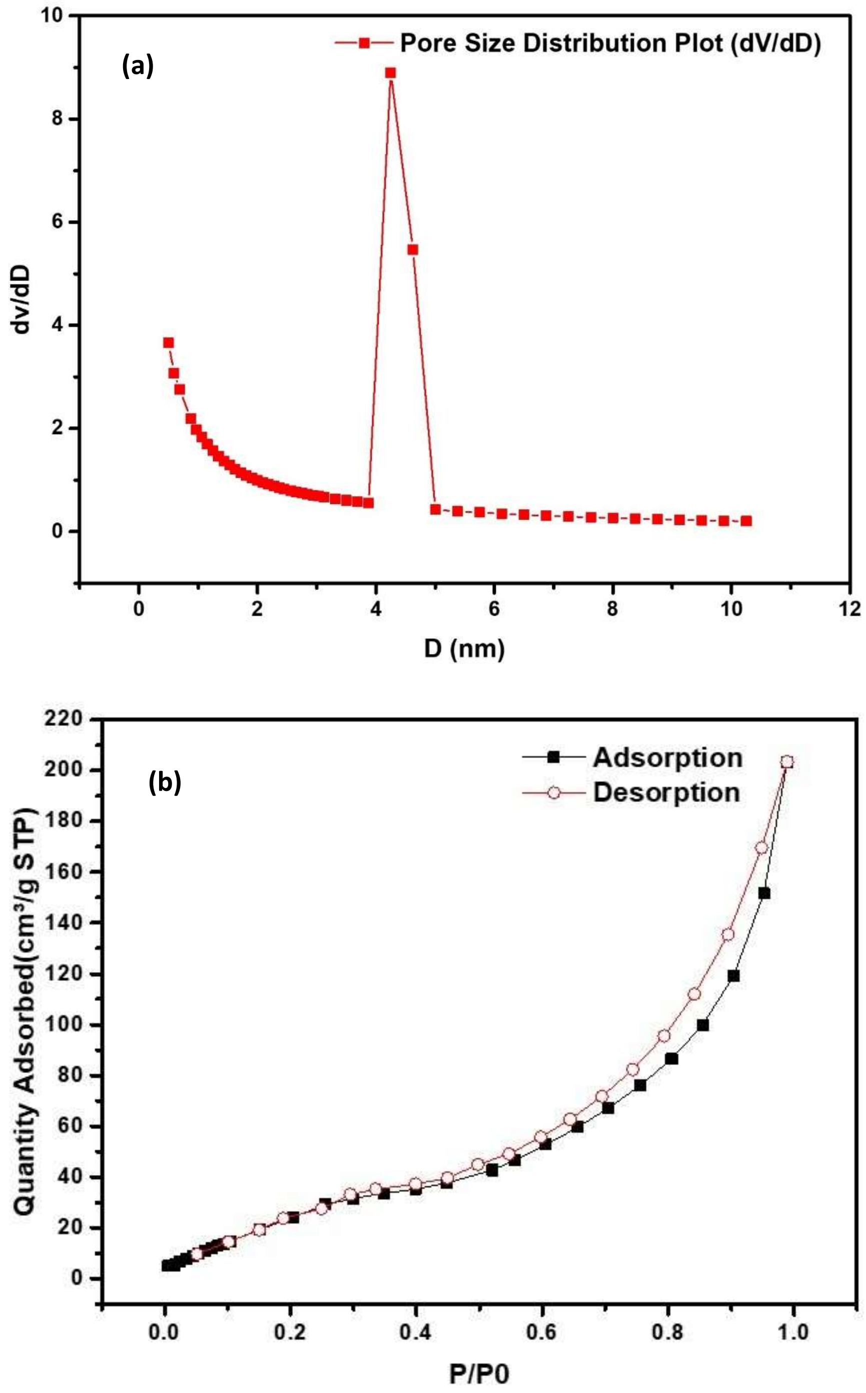

(a)
Pore Size Distribution Plot (dV/dD)
dv/dD
D (nm)
(b)
Adsorption
Desorption
Quantity Adsorbed(cm³/g STP)
P/P0

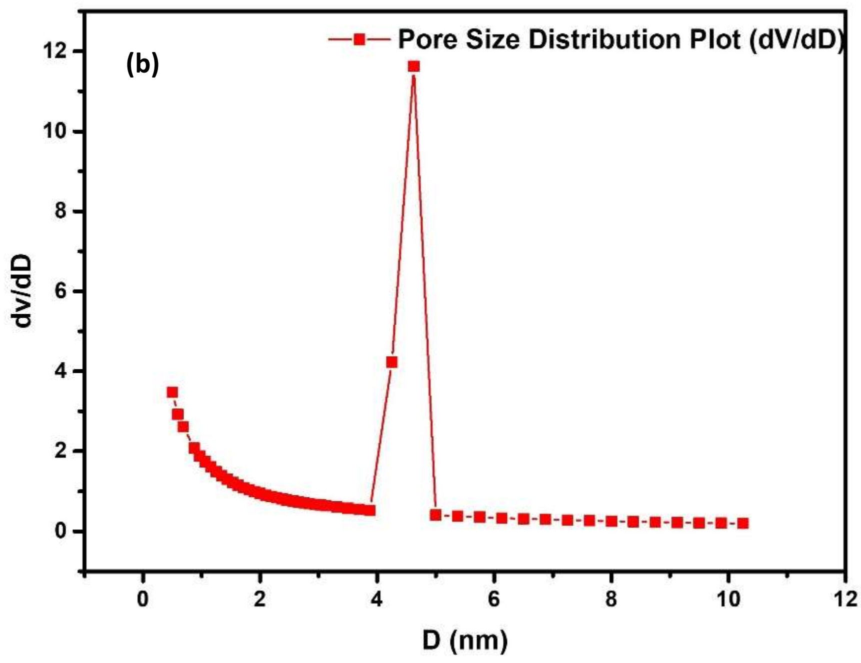

Figure S4. BET surface area analysis results: N_2-adsorption−desorption isotherm plots and the pore size distribution results calculated from the BJH desorption pore volume data for *n*%TCNQ@Sq-1,6Py. a) *n*= 100. b) *n*= 200.

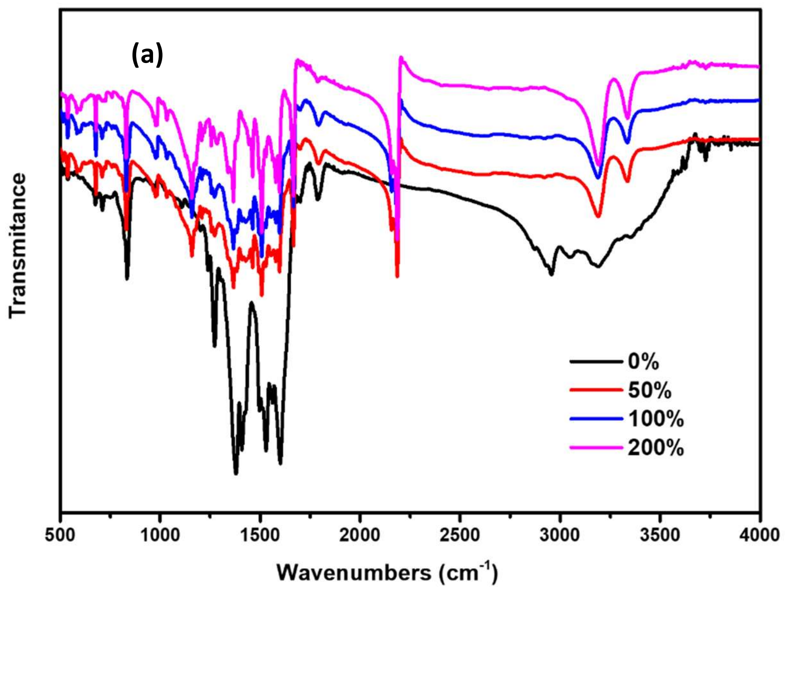

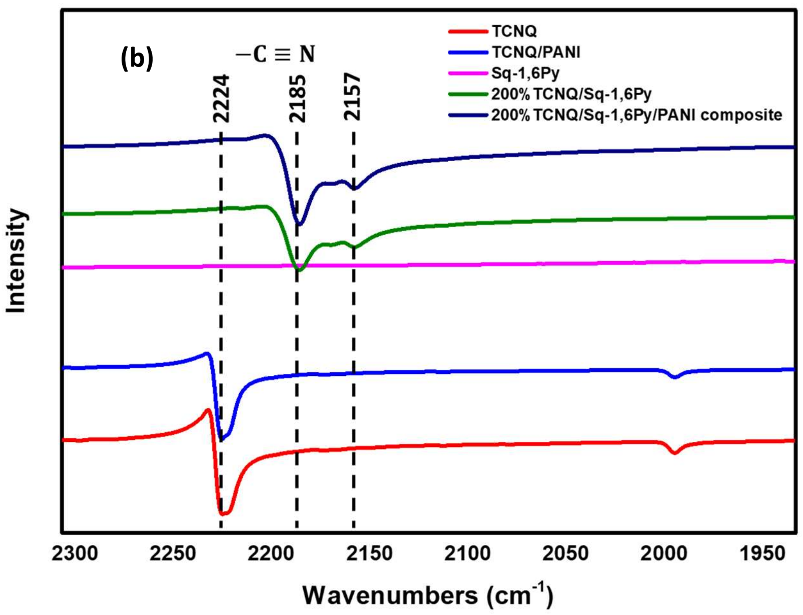

Figure S5. FTIR results of a) *n*%TCNQ@Sq-1,6Py with *n*= 0, 50, 100, and 200. b) magnification of TCNQ, TCNQ/PANI, Sq-1,6Py, *200*%TCNQ@Sq-1,6Py, *200*%TCNQ@Sq.

0%

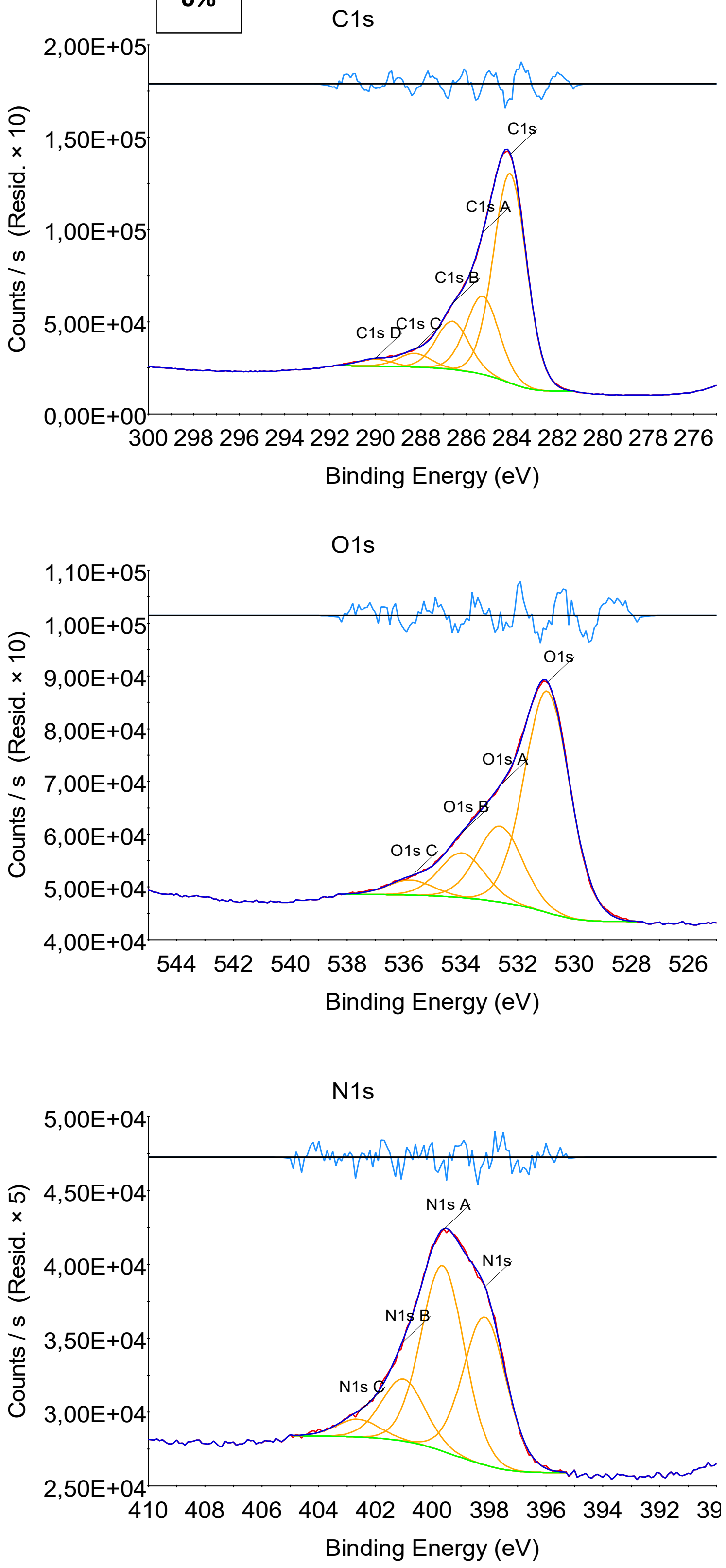

C1s
2,00E+05
1,50E+05
1,00E+05
5,00E+04
0,00E+00
Counts / s (Resid. × 10)
C1s
C1s A
C1s B
C1s C
C1s D
300 298 296 294 292 290 288 286 284 282 280 278 276
Binding Energy (eV)
O1s
1,10E+05
1,00E+05
9,00E+04
8,00E+04
7,00E+04
6,00E+04
5,00E+04
4,00E+04
Counts / s (Resid. × 10)
O1s
O1s A
O1s B
O1s C
544 542 540 538 536 534 532 530 528 526
Binding Energy (eV)
N1s
5,00E+04
4,50E+04
4,00E+04
3,50E+04
3,00E+04
2,50E+04
Counts / s (Resid. × 5)
N1s A
N1s
N1s B
N1s C
410 408 406 404 402 400 398 396 394 392 39
Binding Energy (eV)

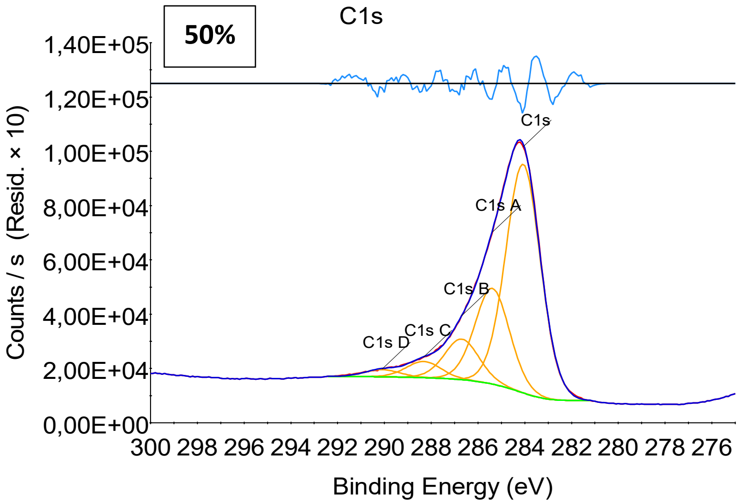
50%
C1s
Counts / s (Resid. × 10)
Binding Energy (eV)
C1s A
C1s B
C1s C
C1s D

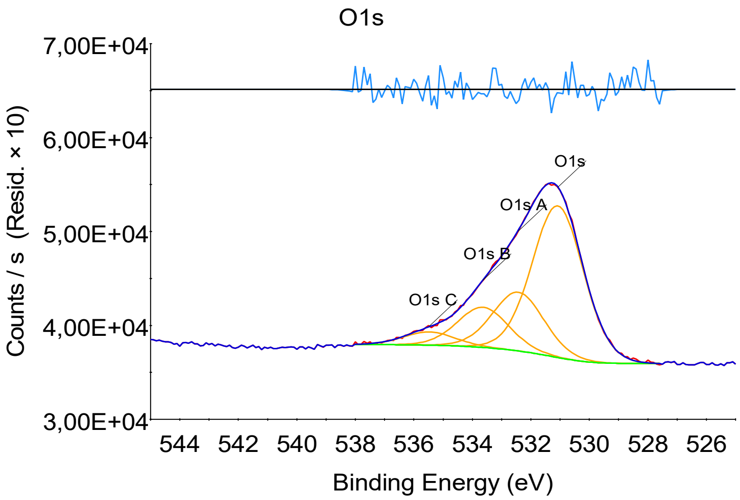
O1s
Counts / s (Resid. × 10)
Binding Energy (eV)
O1s A
O1s B
O1s C

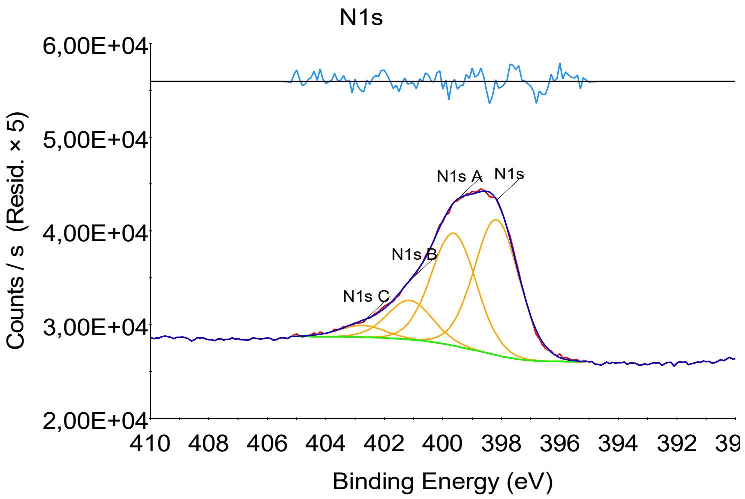
N1s
Counts / s (Resid. × 5)
Binding Energy (eV)
N1s A
N1s B
N1s C

100%

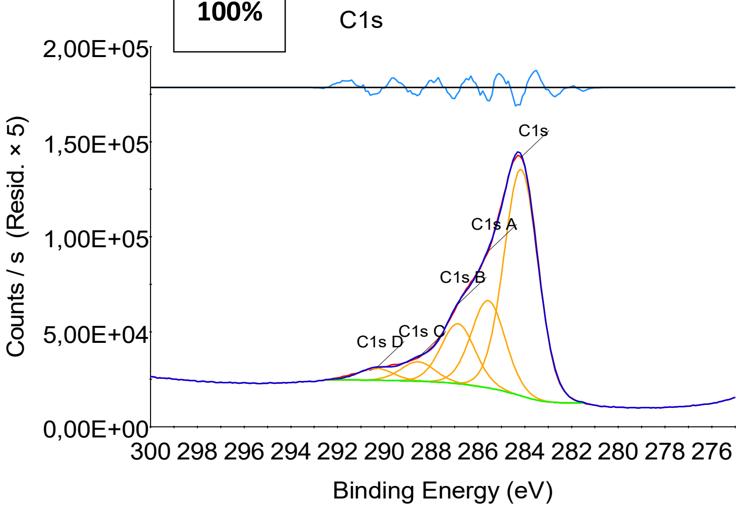
C1s
2,00E+05
1,50E+05
1,00E+05
5,00E+04
0,00E+00
Counts / s (Resid. × 5)
C1s
C1s A
C1s B
C1s C
C1s D
300 298 296 294 292 290 288 286 284 282 280 278 276
Binding Energy (eV)

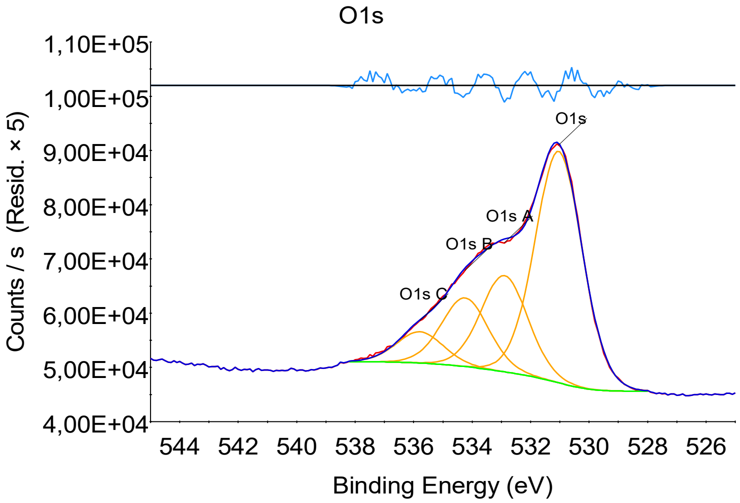
O1s
1,10E+05
1,00E+05
9,00E+04
8,00E+04
7,00E+04
6,00E+04
5,00E+04
4,00E+04
Counts / s (Resid. × 5)
O1s
O1s A
O1s B
O1s C
544 542 540 538 536 534 532 530 528 526
Binding Energy (eV)

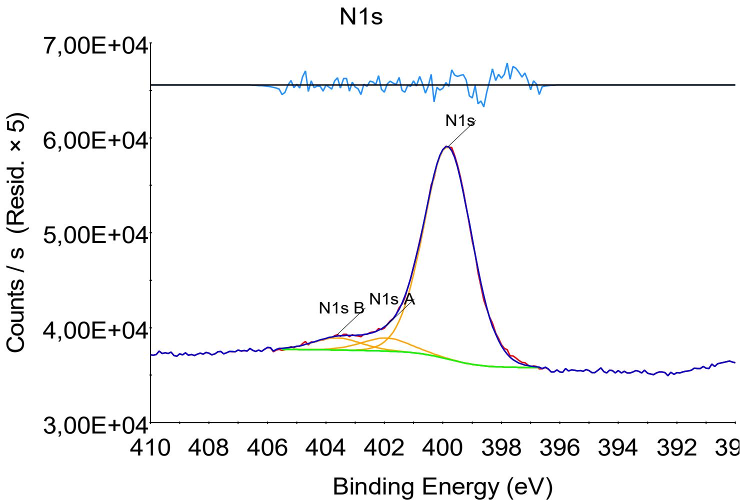
N1s
7,00E+04
6,00E+04
5,00E+04
4,00E+04
3,00E+04
Counts / s (Resid. × 5)
N1s
N1s A
N1s B
410 408 406 404 402 400 398 396 394 392 39
Binding Energy (eV)

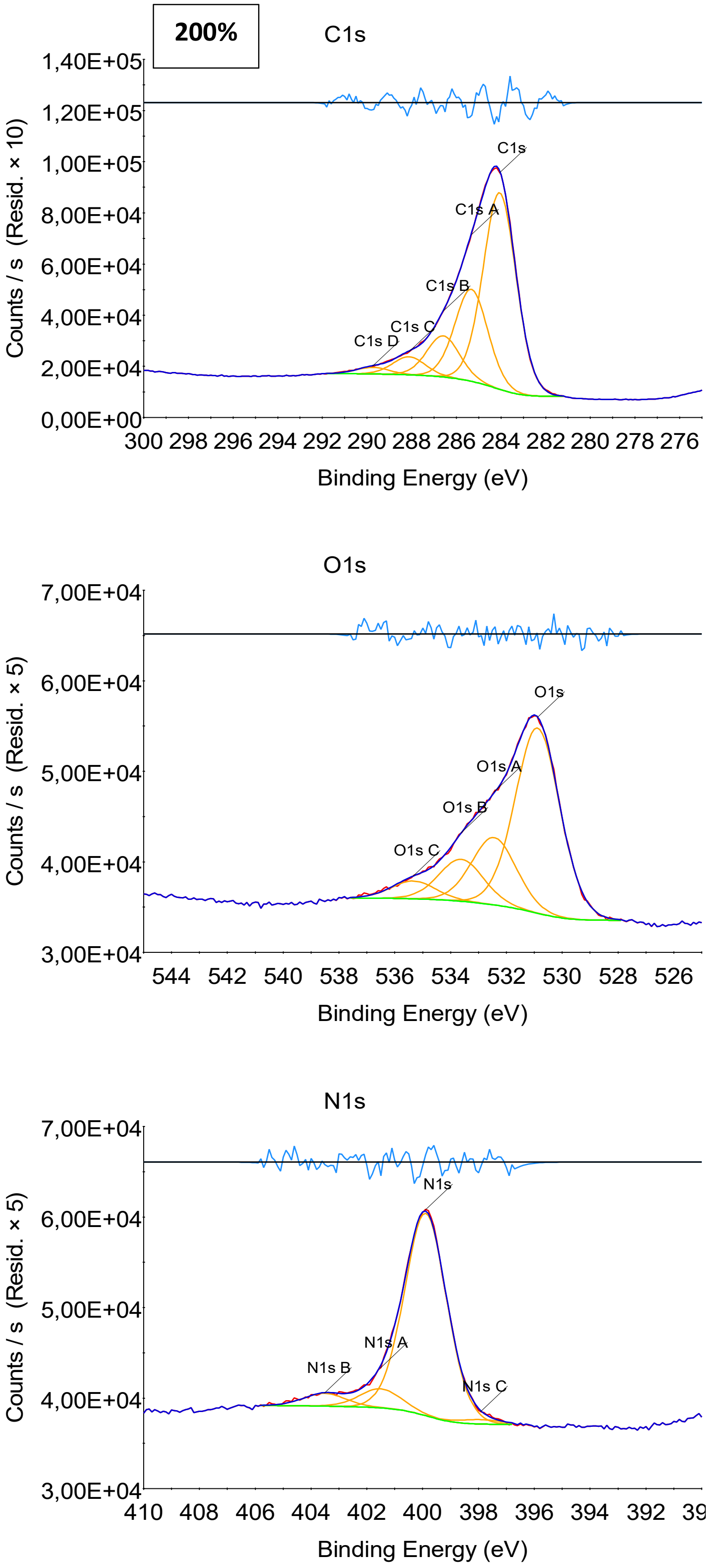

Figure S6. XPS results of *n*%TCNQ@Sq-1,6Py with *n*= 0, 50, 100, and 200.

Table S2. XPS element analysis of *n*%TCNQ@Sq-1,6Py with n= 0, 50, 100, and 200.

CT complex	C (%)	O (%)	N (%)
Sq-1,6Py	83.3	8.33	8.33
50%TCNQ@Sq-1,6Py	81.25	6.25	12.5
100%TCNQ@Sq-1,6Py	80.0	5.0	15.0
200%TCNQ@Sq-1,6Py	78.5	3.5	17.8

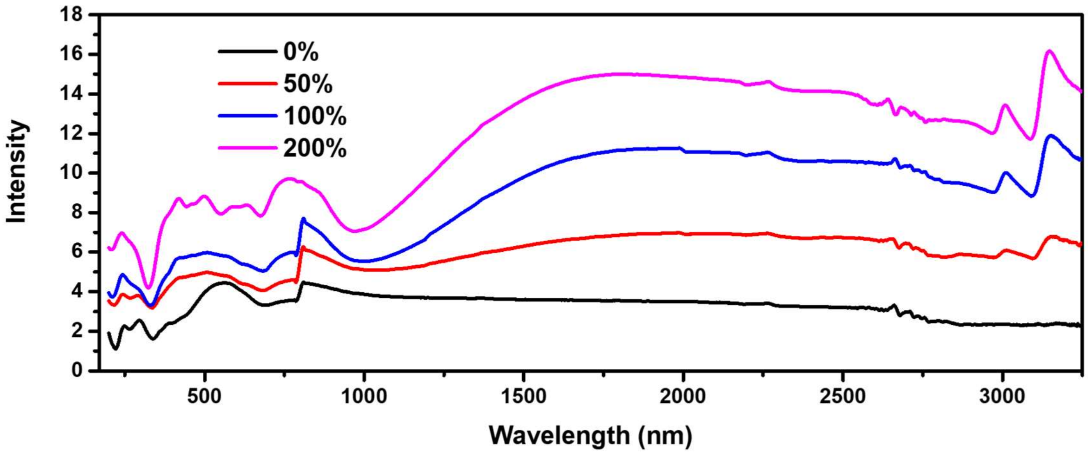

Figure S7. Experimental UV spectrum of *n*%TCNQ@Sq-1,6Py with n= 0, 50, 100, and 200.

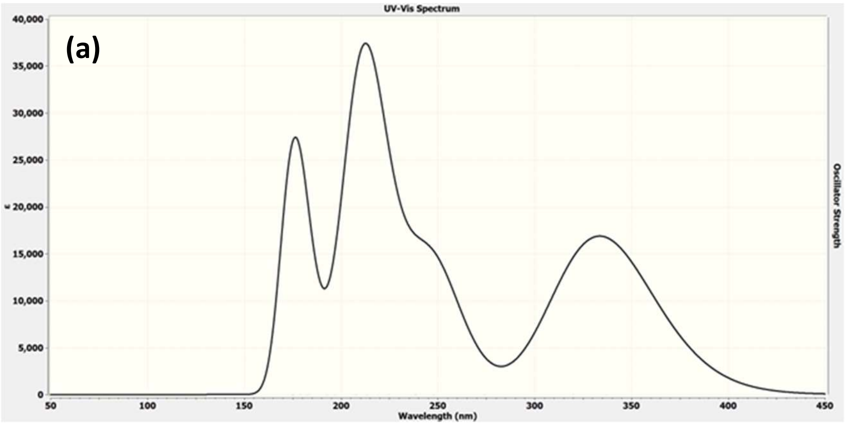

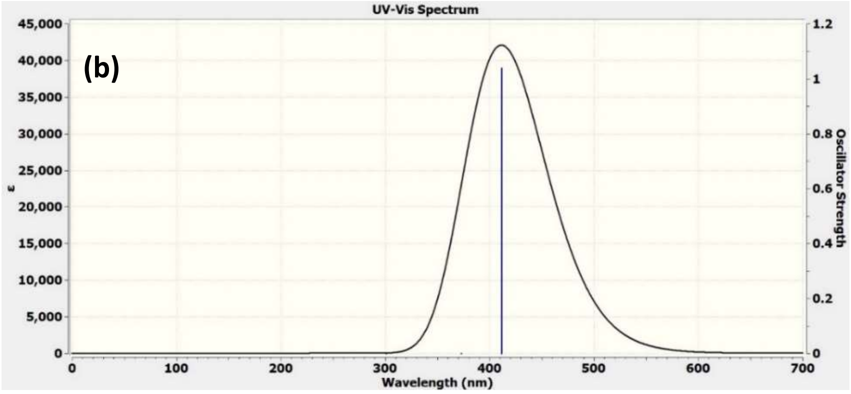

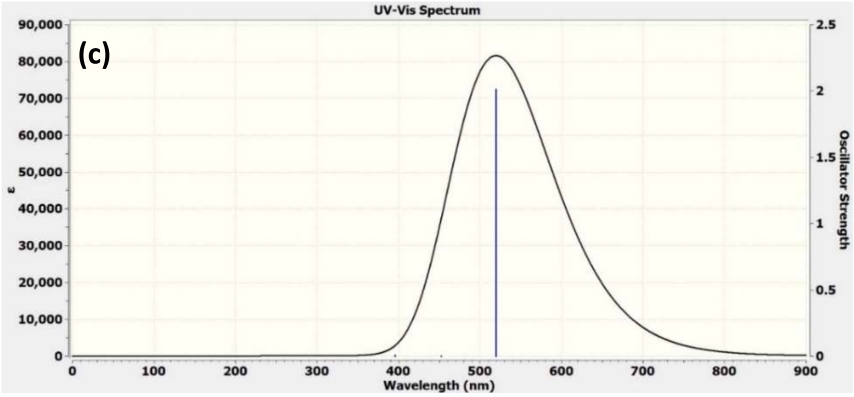

Figure S8. UV spectrum simulation. a) 1,6Py. b) TCNQ. c) Sq-1,6Py.

As a result, 1,6Py cocrystal has higher CT than pyrene, since the energy difference between the HOMO of 1,6Py and the LUMO of TCNQ (0.07295 eV) is less than the difference between the HOMO of pyrene and the LUMO of TCNQ (0.10294 eV) (Figure S9).

LUMO, 1,6Py

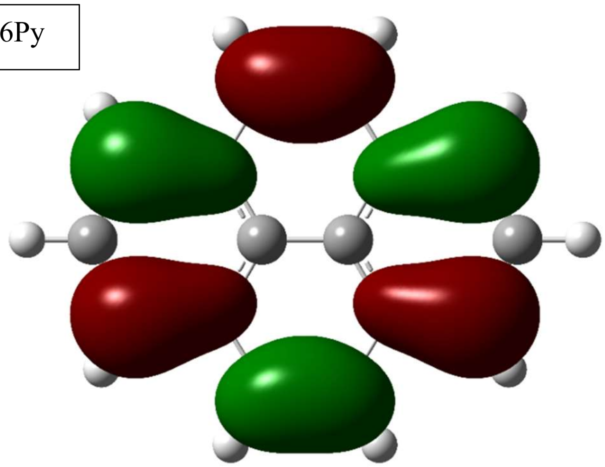

HOMO, 1,6Py

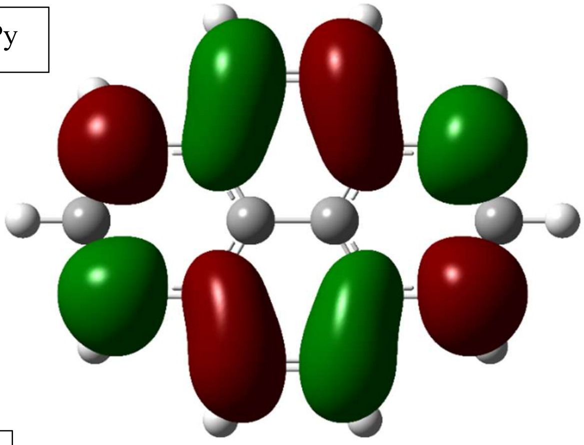

LUMO, TCNQ

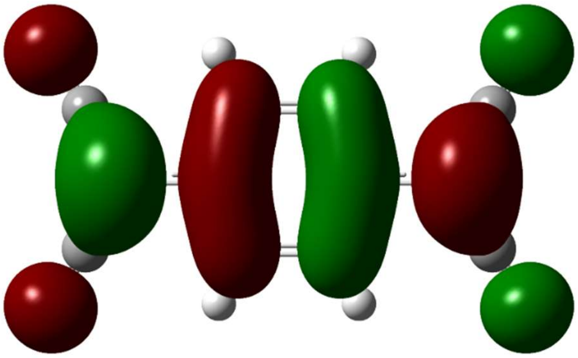

HOMO, TCNQ

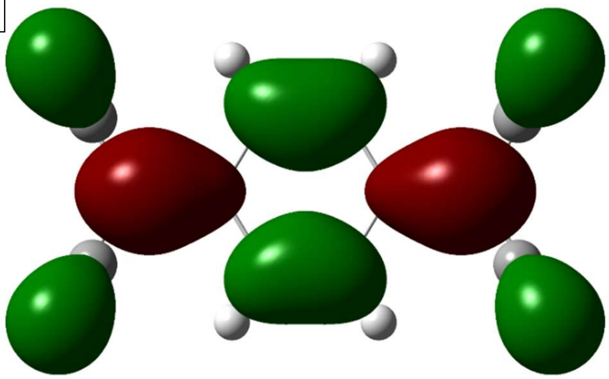

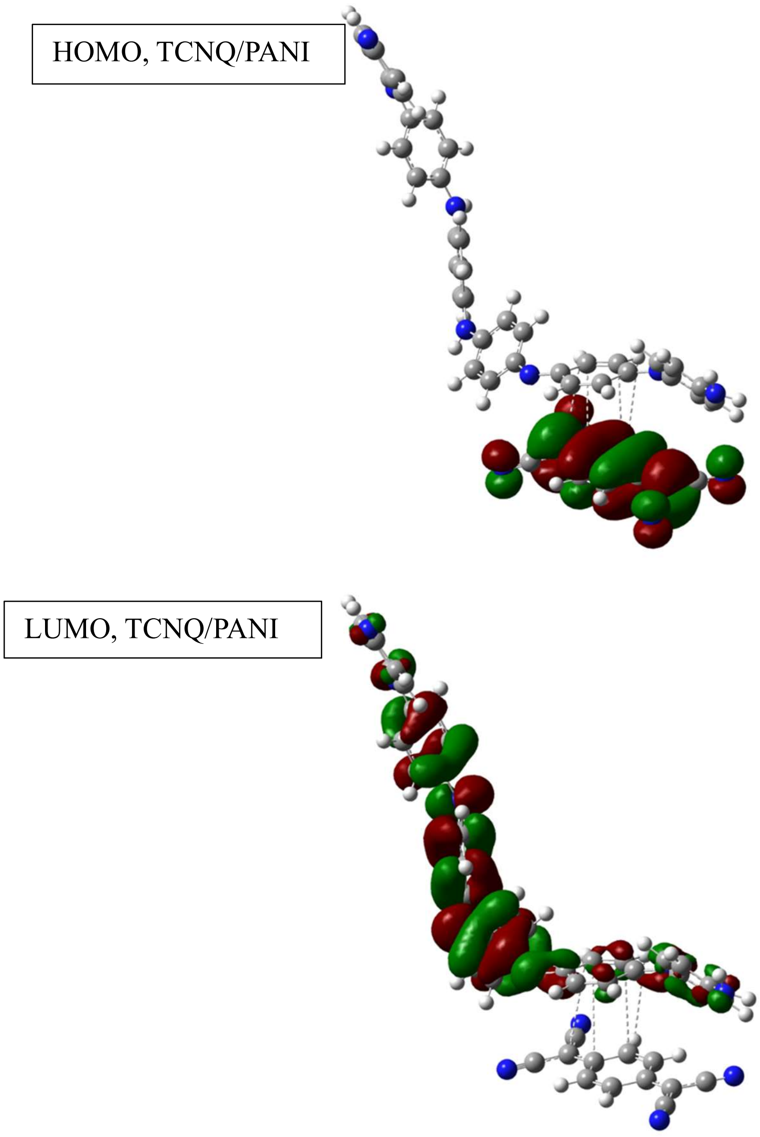

Figure S9. Molecular orbitals; HOMO and LUMO calculation of 1,6Py, TCNQ, and TCNQ/PANI, red and green colors describe the positive and negative phases, respectively.

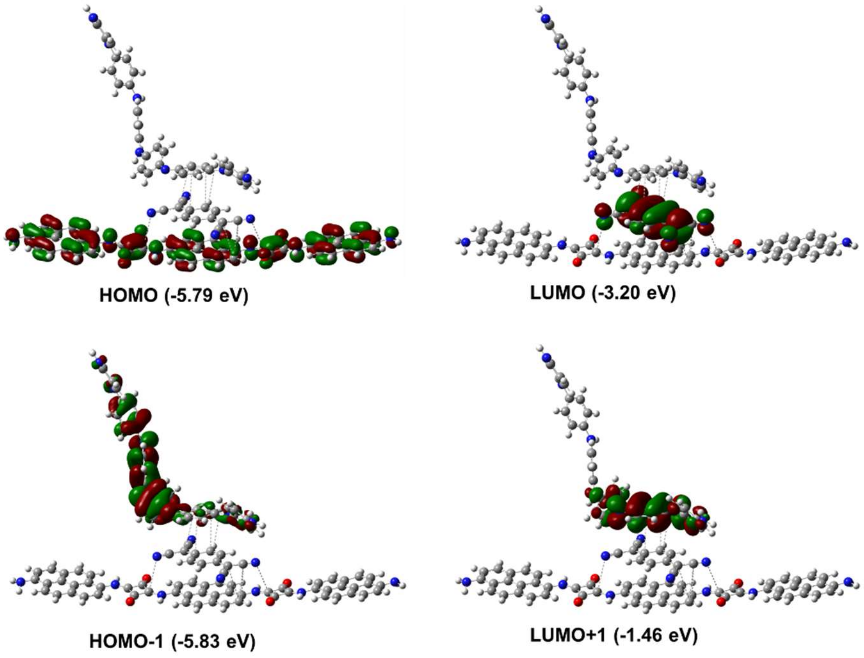

Figure S10. Molecular orbital (HOMO, LUMO, HOMO-1, and LUMO+1) of TCNQ/Sq-1,6Py/PANI composite, red and green colors describe the positive and negative phases, respectively.

To further investigate, the CV curve of TCNQ, PANI, TCNQ/PANI (Figure S11a-c), and the charge impedance of PANI and TCNQ/PANI (Figure S11d) were studied to consider the effect of PANI in their structures. This indicates the inherent electrochemical activity of TCNQ, likely reflecting its charge storage/release characteristics and Sq-1,6Py exhibits a slightly larger current response compared to TCNQ, suggesting enhanced CT kinetics or higher electrochemical activity. In addition, the PANI curve (Figure 11Sb) displays the typical pseudocapacitive behavior with redox peaks corresponding to the reversible redox transitions between its oxidation states and in this regard, broad peaks reflect its capacity for storing charge via faradaic processes.

The obtained data of TCNQ/PANI illustrates R_{ct} value of 7.51 Ω, showing low resistivity rather than PANI and electrical properties probably due to CT reaction between PANI donor and TCNQ acceptor because of activated PANI in H_2SO_4 solution. In addition, the CV curve of TCNQ/PANI proves the acceptable capacitance because the solution atmosphere was changed in H_2SO_4 led to active PANI participation in the CT mechanism. However, in the next step, the electron movement is limited by PANI chains. So, PANI polymers improve the capacitance properties of TCNQ molecules and TCNQ molecules improve the conductivity performance of TCNQ.

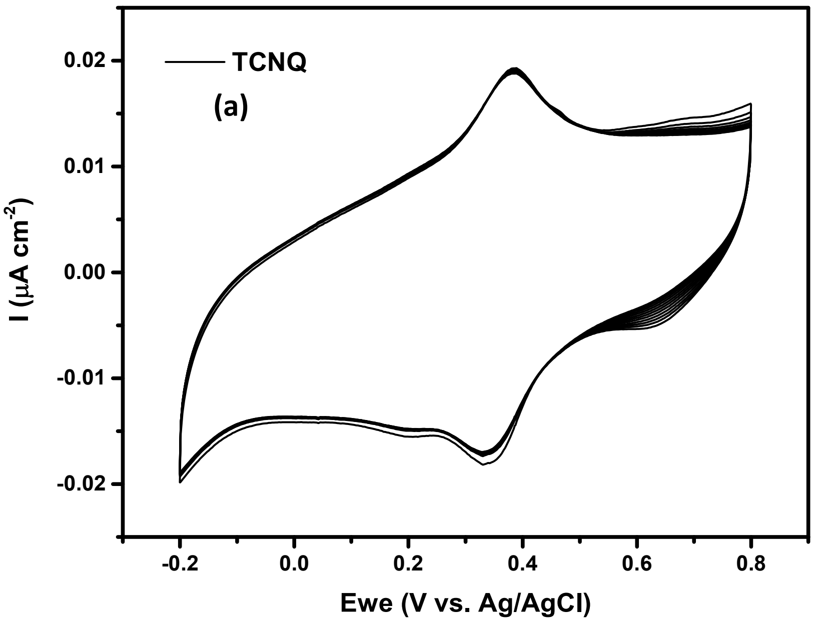
TCNQ
(a)
0.02
0.01
0.00
-0.01
-0.02
I (μA cm-2)
-0.2
0.0
0.2
0.4
0.6
0.8
Ewe (V vs. Ag/AgCl)

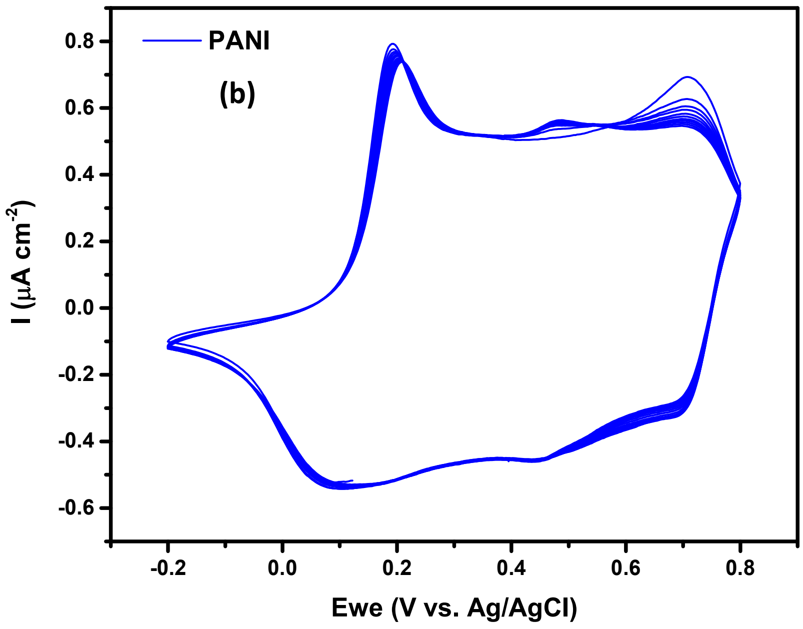
PANI
(b)
0.8
0.6
0.4
0.2
0.0
-0.2
-0.4
-0.6
I (μA cm-2)
-0.2
0.0
0.2
0.4
0.6
0.8
Ewe (V vs. Ag/AgCl)

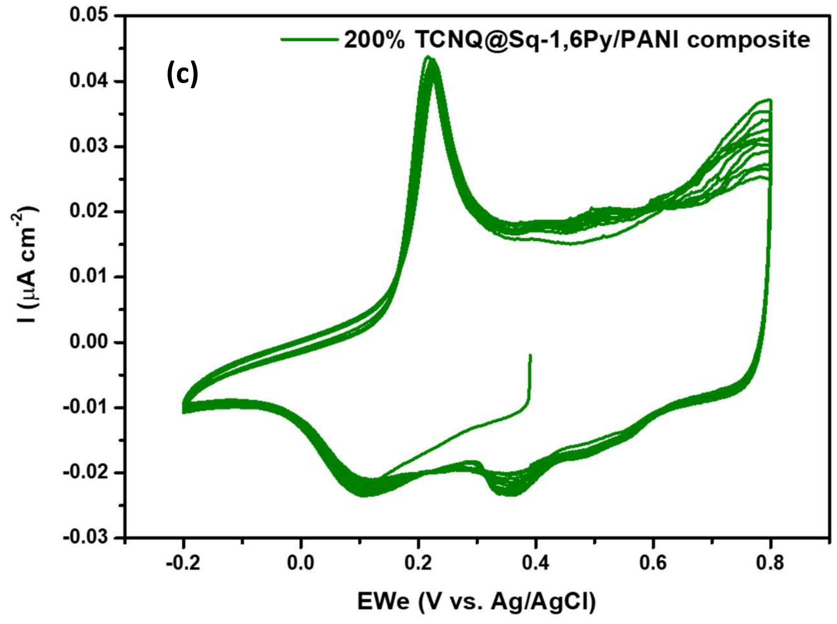
200% TCNQ@Sq-1,6Py/PANI composite
(c)
I (μA cm⁻²)
EWe (V vs. Ag/AgCl)

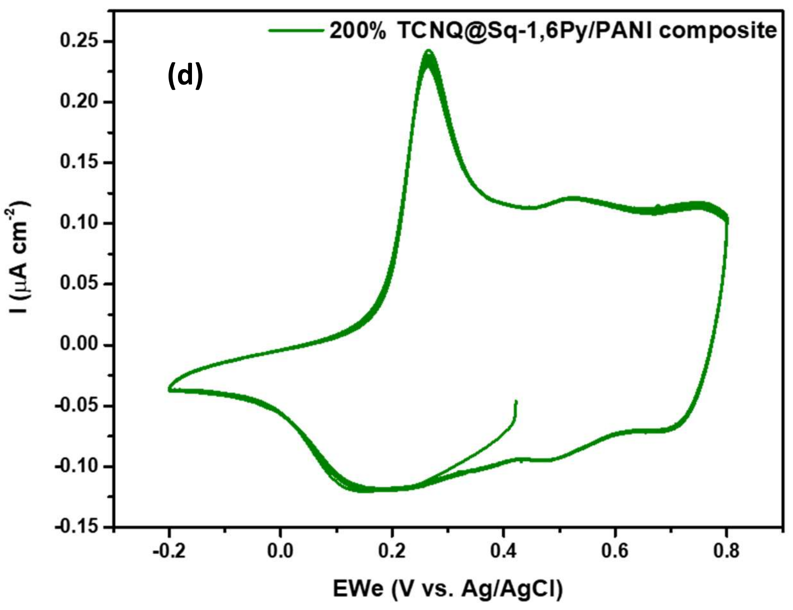
200% TCNQ@Sq-1,6Py/PANI composite
(d)
I (μA cm⁻²)
EWe (V vs. Ag/AgCl)

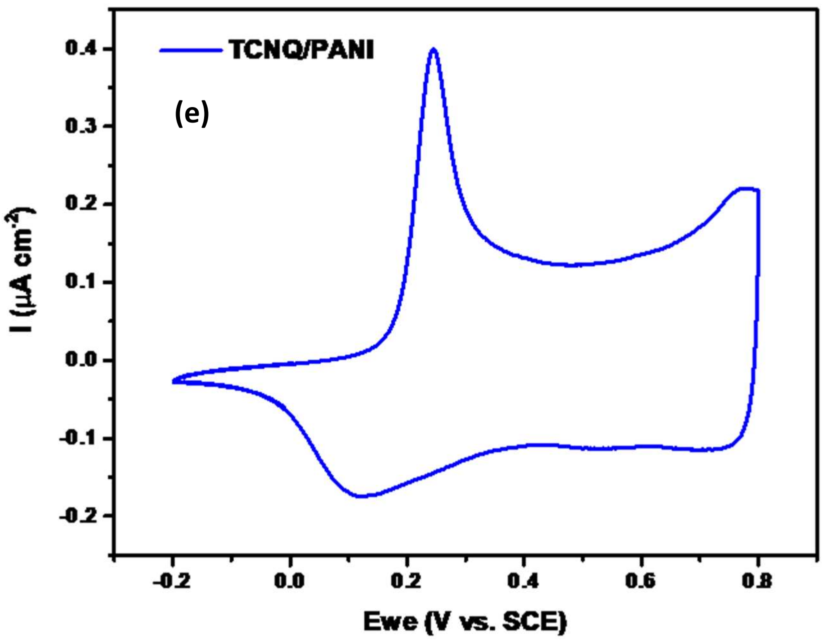
TCNQ/PANI
(e)
I (μA cm-2)
Ewe (V vs. SCE)
0.4
0.3
0.2
0.1
0.0
-0.1
-0.2
-0.2
0.0
0.2
0.4
0.6
0.8

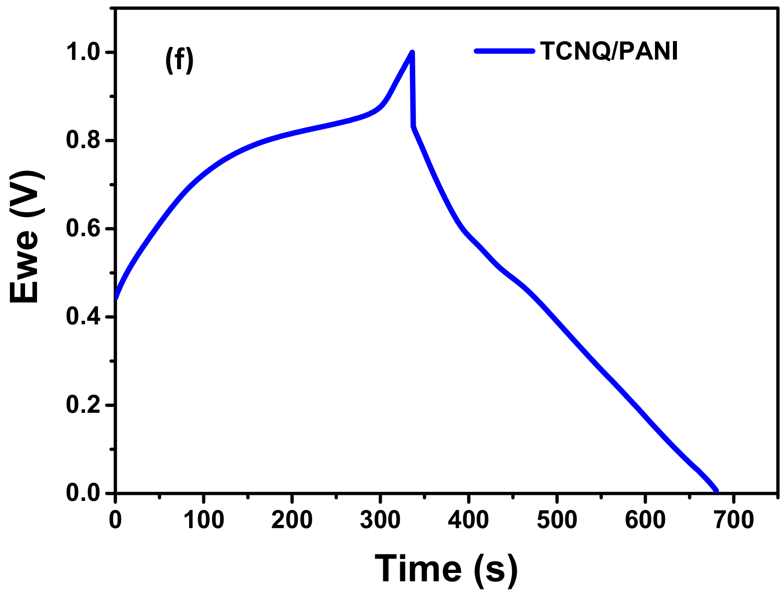
(f)
TCNQ/PANI
Ewe (V)
Time (s)
1.0
0.8
0.6
0.4
0.2
0.0
0
100
200
300
400
500
600
700

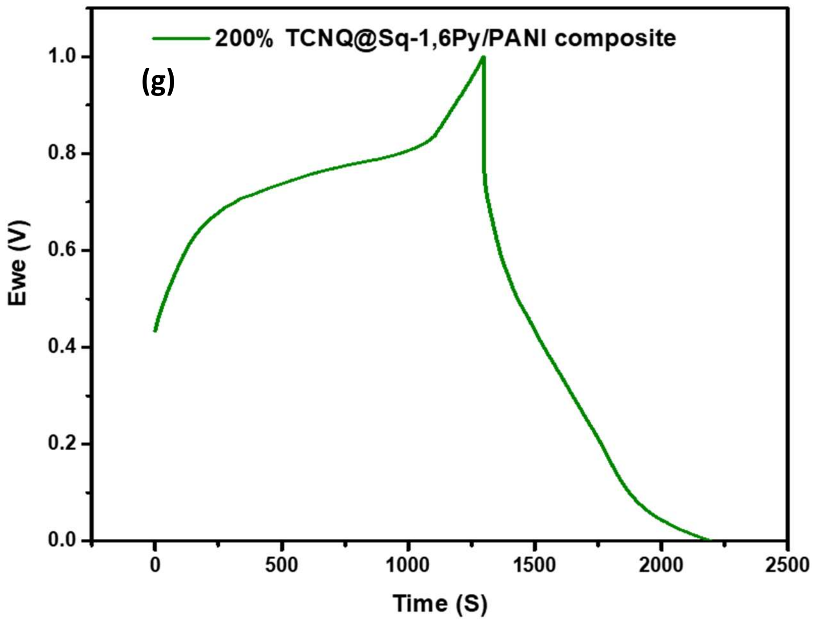
200% TCNQ@Sq-1,6Py/PANI composite
(g)
Ewe (V)
Time (S)
0.0
0.2
0.4
0.6
0.8
1.0
0
500
1000
1500
2000
2500

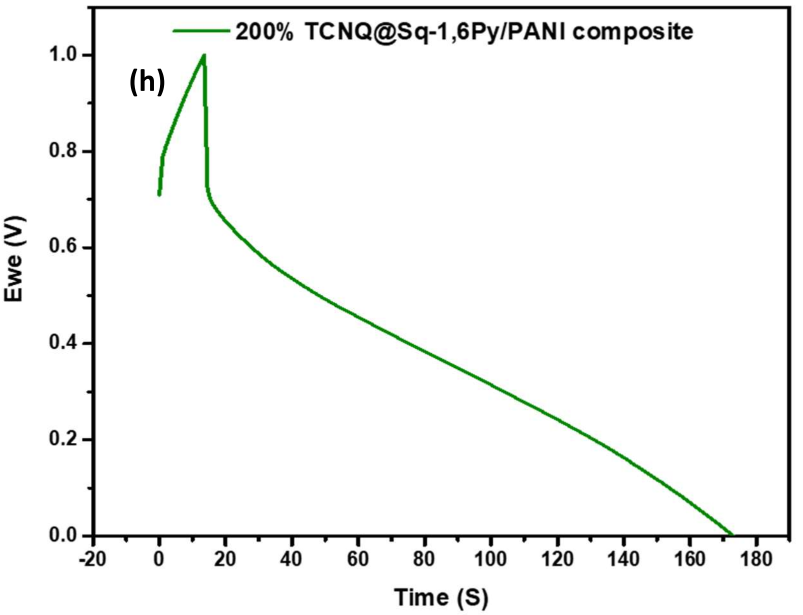
200% TCNQ@Sq-1,6Py/PANI composite
(h)
Ewe (V)
Time (S)
0.0
0.2
0.4
0.6
0.8
1.0
-20
0
20
40
60
80
100
120
140
160
180

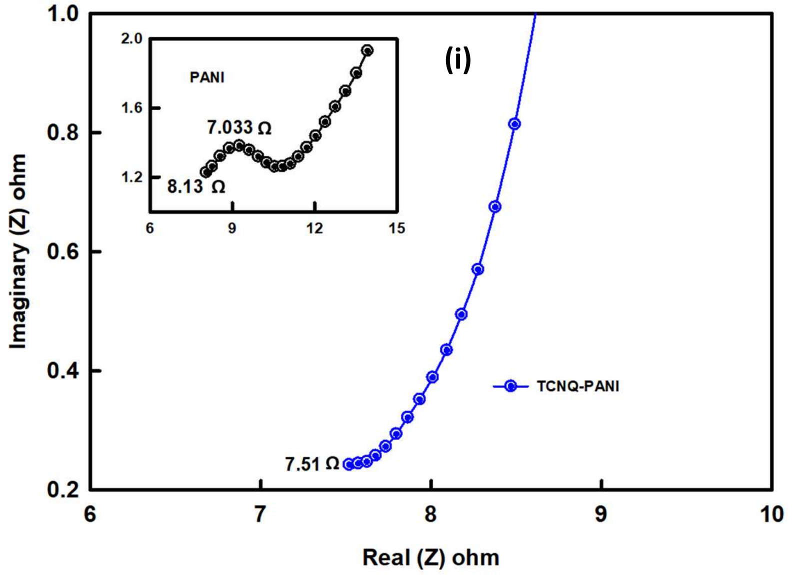

Figure S11. Electrochemical display of the electrodes in a three-electrode system in the aqueous electrolyte (0.5 M H_2SO_4), a) C-V curve of TCNQ. b) C-V curve of PANI. c) C-V curve of *200*%TCNQ@Sq-1,6Py/PANI composite at 10 mV^{-1} scan rate. d) C-V curve of *200*%TCNQ@Sq-1,6Py/PANI composite at 80 mV^{-1} scan rate. e) C-V curve of TCNQ/PANI at 10 mV^{-1}. f) Charge–discharge profile of TCNQ/PANI with a current density of 0.312 A g^{-1}. g) Charge–discharge profile of *200*%TCNQ@Sq-1,6Py/PANI composite with a current density of 0.285 A g^{-1}. h) Charge–discharge profile of *200*%TCNQ@Sq-1,6Py/PANI composite with a current density of 0.75 A g^{-1}. i) Nyquist impedance plots of PANI and TCNQ/PANI.

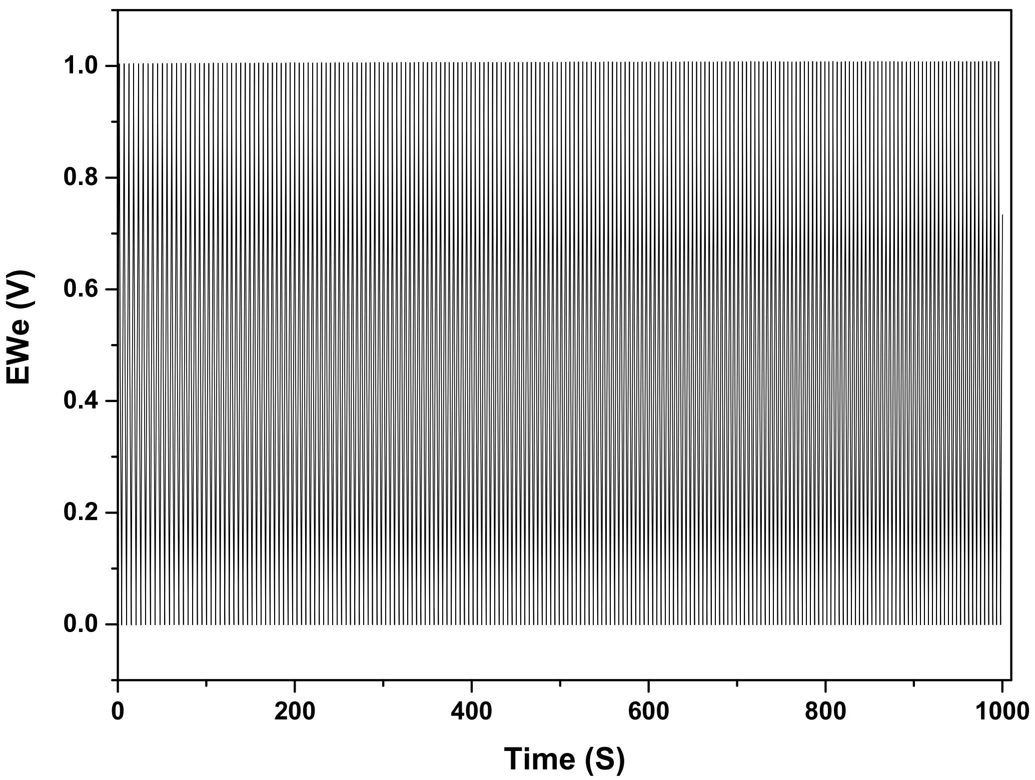
EWe (V)
Time (S)
1.0
0.8
0.6
0.4
0.2
0.0
0
200
400
600
800
1000

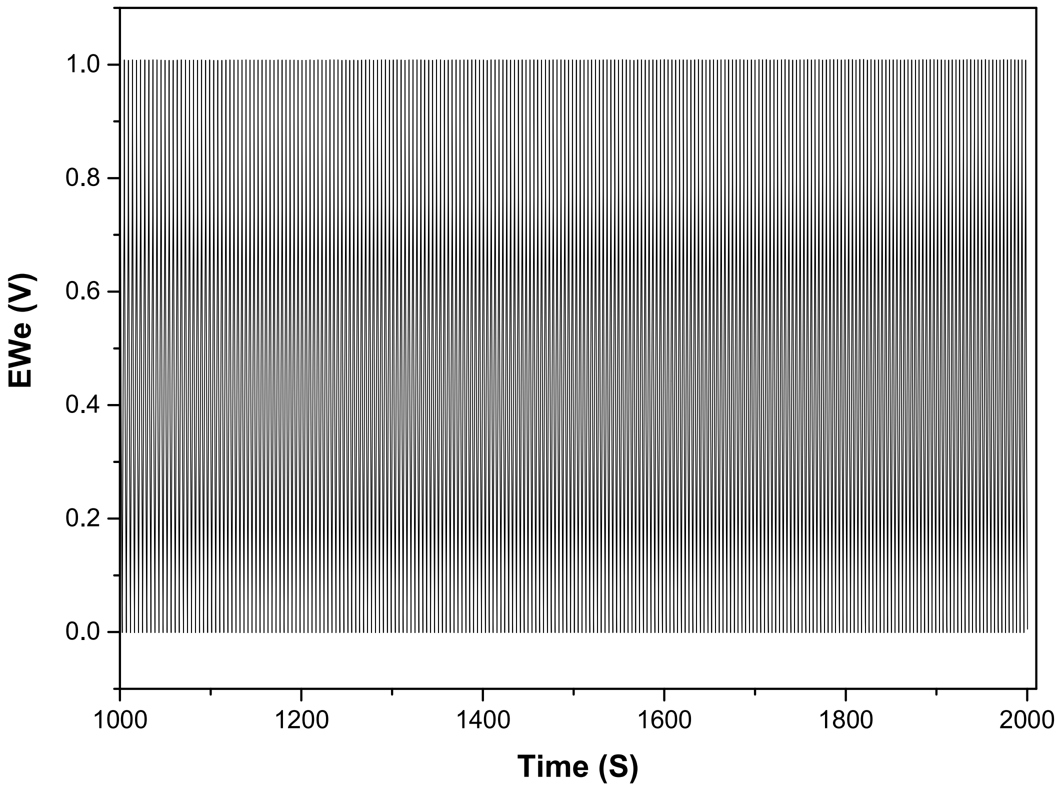
EWe (V)
Time (S)
1.0
0.8
0.6
0.4
0.2
0.0
1000
1200
1400
1600
1800
2000

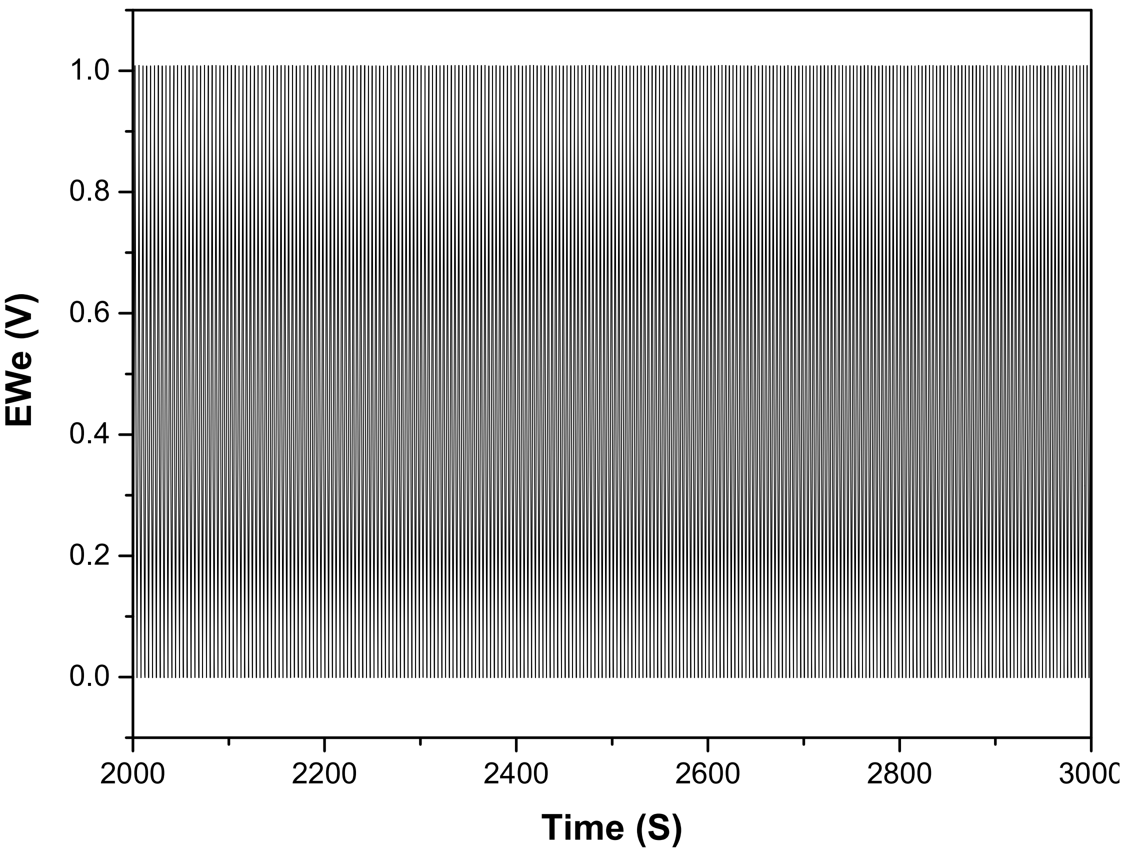
EWe (V)
Time (S)
1.0
0.8
0.6
0.4
0.2
0.0
2000
2200
2400
2600
2800
3000

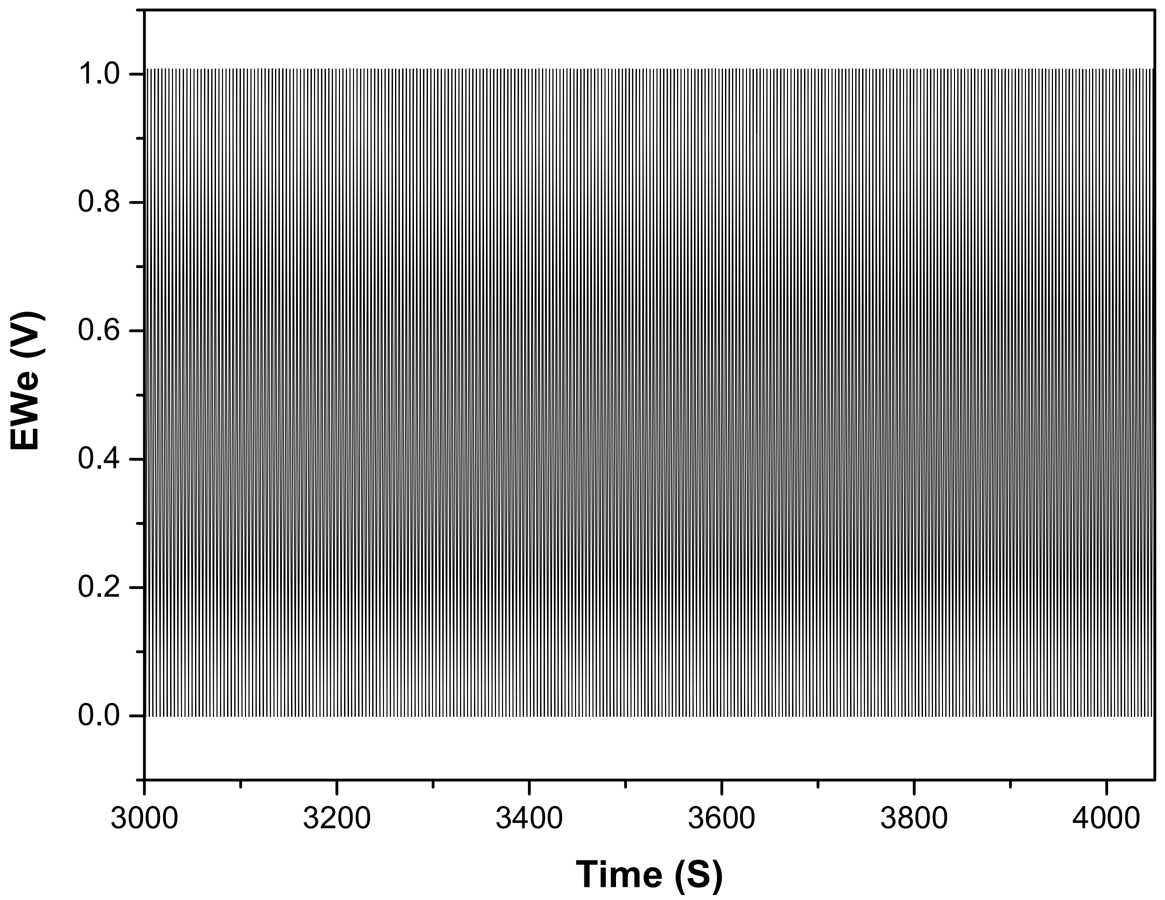

Figure S12. Cycling stability of *200*%TCNQ@Sq-1,6Py/PANI composite for 1000 charge-discharge cycles.

Furthermore, the results of EIS under UV light (365 nm) confirmed the decreasing R_s (2.81, 2.74, 2.48, and 2.31 Ω) and R_{ct} (3.47, 2.64, 2.06, and 1.82 Ω) until 60 min for *200*%TCNQ@Sq-1,6Py and *200*%TCNQ@Sq-1,6Py/PANI composite, respectively. The results of heating showed a little decreasing R_s (2.81 Ω at 25 ℃, 2.58 Ω at 40 ℃, 2.38 Ω at 60 ℃, 2.15 Ω at 80 ℃) and the R_{ct} (3.47 Ω at 25 ℃, 4.70 Ω at 40 ℃, 4.24 Ω at 60 ℃, 3.53 Ωat 80 ℃) with the reducing rate range (0.07-0.09) for *200*%TCNQ@Sq-1,6Py and *200*%TCNQ@Sq-1,6Py/PANI composite, respectively (Figure S13).

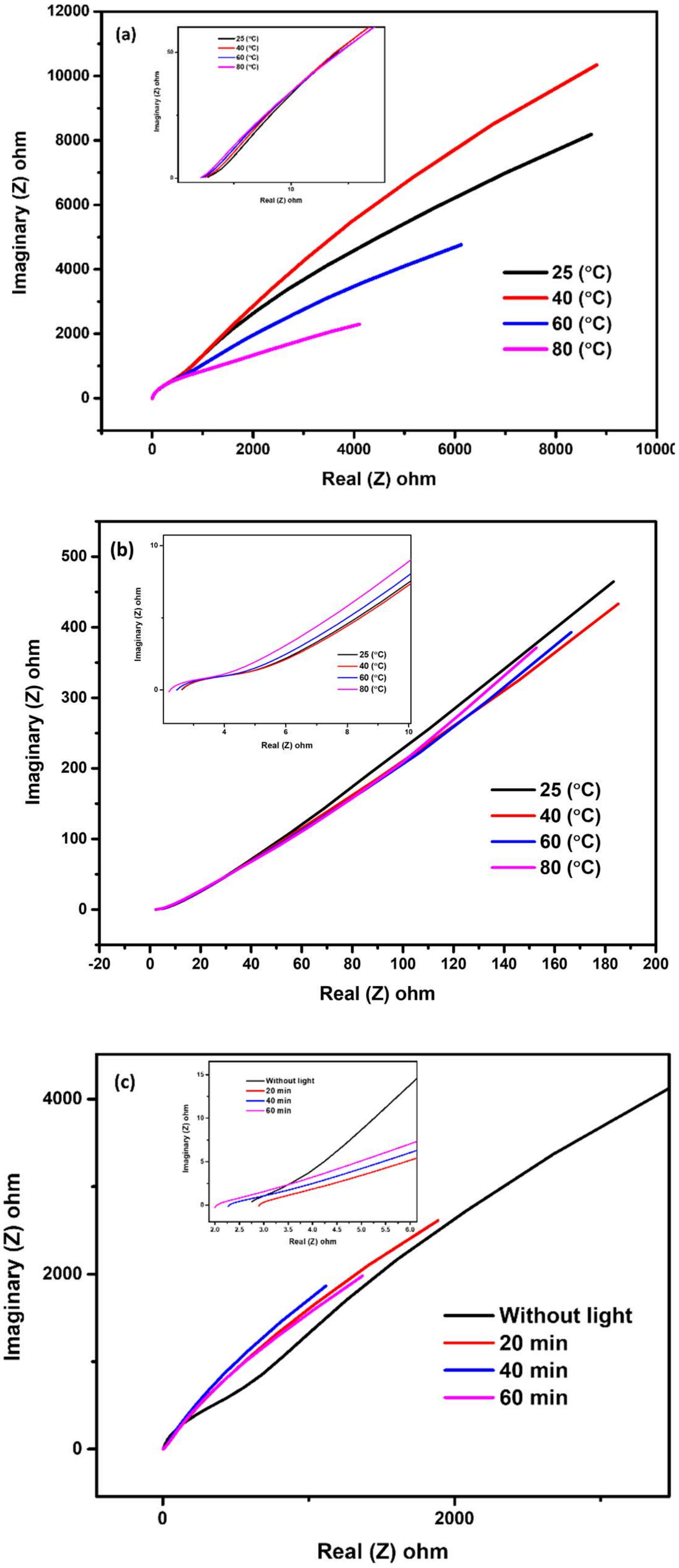
(a)
Imaginary (Z) ohm
Real (Z) ohm
25 (°C)
40 (°C)
60 (°C)
80 (°C)
(b)
Imaginary (Z) ohm
Real (Z) ohm
25 (°C)
40 (°C)
60 (°C)
80 (°C)
(c)
Imaginary (Z) ohm
Real (Z) ohm
Without light
20 min
40 min
60 min

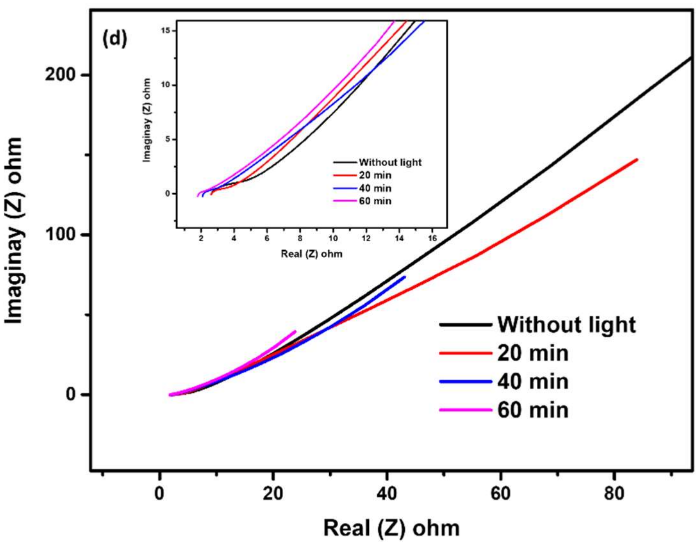

Figure S13. Electrochemical display of the electrodes in a three-electrode system in the aqueous electrolyte (0.5 M H_2SO_4), Nyquist impedance plots of a) *200*%TCNQ@Sq-1,6Py at different temperatures. b) *200*%TCNQ@Sq-1,6Py/PANI composite at different temperatures. c) *200*%TCNQ@Sq-1,6Py under UV light. d) *200*%TCNQ@Sq-1,6Py/PANI under UV light.

Remarkably, the electrical conductivity of *200*%TCNQ@Sq-1,6Py (8.7×10^{-2} S cm^{-1}) is comparable to that of many reported organic materials and even organic-metal materials, such as TTF-TCNQ/LiTFSI (10^{-8} S cm^{-1}) [2], [$C_6H_2(NH_2)_4$][TCNQ] (9.8×10^{-4} S cm^{-1}) [3], [$C_6H_2(NH_2)_4$][F_4TCNQ] (2.8×10^{-5} S cm^{-1}) [3], [$Rb^+$$(TCNQ)^-$] ($10^{-2}$ S cm^{-1}), TCNQ@HKUST-1 (2.42×10^{-2} S cm^{-1}) [4], [T1(TCNQCl_2)] phase I (9.3×10^{-3} S cm^{-1}) [5], and [T1(TCNQBr_2)] (2.1×10^{-3} S cm^{-1}) [5] (Figure S14).

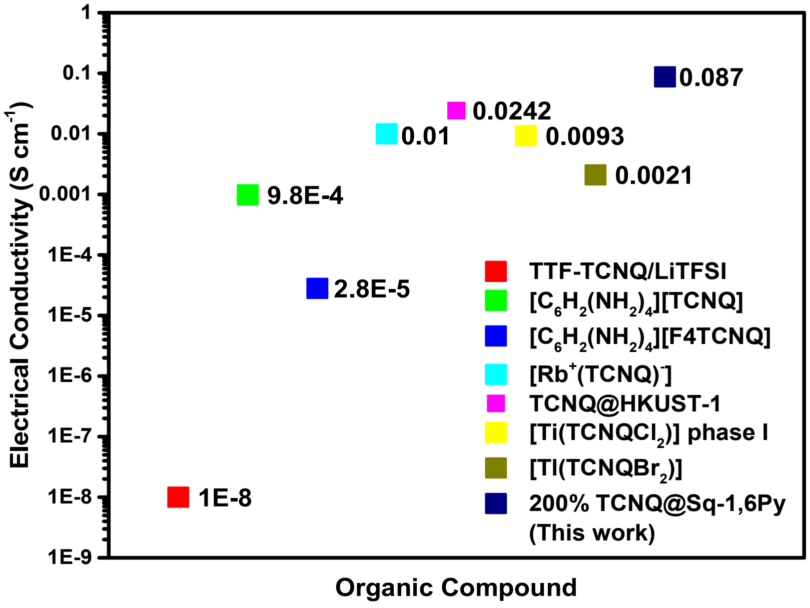

Figure S14. Electrical conductivity amounts of some organic compounds based on the literature.

Table S3. DFT method, UV-vis, and CV bandgap values of CT complexes.

Name	DFT band gap (eV)	Uv-vis band gap (eV)	CV band gap (eV)
0%TCNQ@Sq-1,6Py	1.46	1.47	1.51
50%TCNQ@Sq-1,6Py	1.34	1.37	1.40
100%TCNQ@Sq-1,6Py	1.25	1.30	1.37
200%TCNQ@Sq-1,6Py	1.12	1.24	1.38
PANI	---	----	2.95

References

[1] a) K. Ajeel, Q. Kareem, in *Journal of Physics: Conference Series* IOP Publishing, **2019**, 012020; b) B. S. Singu, P. Srinivasan, S. Pabba, *Journal of The Electrochemical Society* **2011**, *159* (1), A6.

[2] K. Hatakeyama-Sato, M. Umeki, T. Tezuka, K. Oyaizu, *ACS Applied Electronic Materials* **2020**, *2* (7), 2211.

[3] A. L. Sutton, B. F. Abrahams, D. M. D'Alessandro, L. Goerigk, T. A. Hudson, R. Robson, P. M. Usov, *Journal of Materials Chemistry C* **2020**, *8* (27), 9422.

[4] S. Jung, L. Huelsenbeck, Q. Hu, S. Robinson, G. Giri, *ACS Applied Materials & Interfaces* **2021**, *13* (8), 10202.

[5] Z. Zhang, H. Zhao, H. Kojima, T. Mori, K. R. Dunbar, *Chemistry–A European Journal* **2013**, *19* (10), 3348.
